# Lactate Activates the HCAR1/β‐Arrestin2/PP2A Signaling Axis to Mediate STAT1/2 Dephosphorylation and Drive Osteosarcoma Progression

**DOI:** 10.1002/advs.202506214

**Published:** 2025-09-16

**Authors:** Zhitao Han, Qi Jia, Guangjian Bai, Zhao Li, Jin Zeng, Zhenhua Wang, Jing Zhang, Jiashi Cao, Baoquan Xin, Yonggang Liu, Jingyu Shen, Lining Wang, Xing Huang, Chunlei Zhang, Kai Tong, Fenglei Dai, Yajie Zhang, Weina Zhu, Songyi Cheng, Yuanming Chen, Xinghai Yang, Guanghui Chen, Zhenhua Zhou, Yong Ma, Weibo Liu, Ting Wang, Tielong Liu

**Affiliations:** ^1^ School of Integrated Chinese and Western Medicine Nanjing University of Chinese Medicine Nanjing Jiangsu 210023 China; ^2^ Nanjing Hospital of Chinese Medicine Affiliated to Nanjing University of Chinese Medicine Nanjing Jiangsu 210022 China; ^3^ Department of Orthopaedic Oncology The Second Affiliated Hospital of Naval Medical University Shanghai 200003 China; ^4^ School of Health Science and Engineering University of Shanghai for Science and Technology Shanghai 200090 China; ^5^ Department of Orthopedics The First Affiliated Hospital of Nanchang University Nanchang Jiangxi 330006 China; ^6^ Department of Centre for Translational Medicine The Second Affiliated Hospital of Naval Medical University Shanghai 200003 China; ^7^ Oncology Department Kunshan Hospital of Chinese Medicine Suzhou Jiangsu 215301 China; ^8^ Department of Orthopedics Shanghai Pudong Hospital Fudan University Pudong Medical Center Shanghai 200127 China; ^9^ Central Laboratory Nanjing Hospital of Chinese Medicine Affiliated to Nanjing University of Chinese Medicine Nanjing Jiangsu 210022 China; ^10^ Department of Orthopedics Second Affiliated Hospital of Guangxi Medical University Nanning Guangxi 530005 China; ^11^ Department of Orthopaedics Peking University Third Hospital Beijing 100191 China; ^12^ Yancheng TCM Hospital Affiliated to Nanjing University of Chinese Medicine Yancheng TCM Hospital Yancheng Jiangsu 224002 China; ^13^ Jiangsu CM Clinical Innovation Center of Degenerative Bone & Joint Disease Wuxi TCM Hospital Affiliated to Nanjing University of Chinese Medicine Wuxi Jiangsu 214071 China; ^14^ Department of Orthopaedics The Fourth Medical Centre Chinese PLA General Hospital Beijing 100853 China

**Keywords:** β‐Arrestin 2, HCAR1, lactate, osteosarcoma, STAT1/2

## Abstract

The “Warburg effect”, a hallmark of Osteosarcoma(OS), results in lactate accumulation due to aerobic glycolysis. The role and underlying mechanisms of lactate in OS are not well understood. Herein, the lactate‐activated hydroxycarboxylate receptor 1(HCAR1) is found to promote OS progression via inhibiting the transcription of anti‐oncogene downstream of STAT1/2. The phosphorylation level of STAT1/2 holds considerable significance for transcriptional activity. In this study, protein phosphatase 2A(PP2A) is identified as the tyrosine phosphatase of STAT1/2. Lactate‐activated HCAR1, facilitating PP2A interaction with phosphorylated STAT1/2 via β‐Arrestin 2, resulting in STAT1/2 dephosphorylation, a key process linked to the aggressive behavior of OS. Using PP2A inhibitor Endothall can abolish the dephosphorylation effect of HCAR1 on STAT1/2, inhibit cancer cell proliferation, migration, and cell cycle, and promote apoptosis. Moreover, the combination of Endothall and Cisplatin is high synergistic in treating OS. In conclusion, the study elucidates the pro‐oncogenic role of lactate‐activated HCAR1 in OS.

## Graphical abstract

In the present study, it is found that in osteosarcoma, lactate‐activated HCAR1 promotes β‐arrestin 2 translocation from cytoplasm to nucleus. In the nucleus, β‐arrestin 2 recruits PP2A to the activated STAT1/2 dimers, mediating STAT1/2 dephosphorylation and inhibiting the transcription of downstream anti‐oncogenes to promote OS progression.

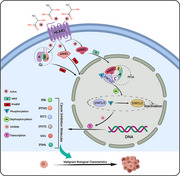

## 1 Introduction

OS is the most common primary bone malignancy in children and adolescents,^[^ ^1^ ^]^ often occurring in the long bones and with a poor prognosis.^[^ ^2^ ^]^ The etiology for OS remains elusive, as there is no single driver gene mutation responsible for its development. Instead, numerous genetic alterations have been associated with OS. This extensive genetic and epigenetic heterogeneity of OS complicates the identification and development of new therapeutic strategies. OS is highly heterogeneous among different patients, and even the same patient also experiences tumors heterogeneous changes before and after chemotherapy. However, OS consistently exhibits aerobic glycolysis, a metabolic trait that is retained regardless of genetic and epigenetic variations.

Even in oxygen‐rich environments, cancer cells present enhanced glucose consumption and lactate production, known as the “Warburg effect”.^[^ ^3^ ^]^ Aerobic glycolysis, despite its lower ATP yield compared to oxidative phosphorylation, provides cancer cells with several advantages through the production of lactate. Lactate acts as a substrate for the tricarboxylic acid cycle, sustaining energy supply within the tumor microenvironment.^[^ ^4^ ^]^ Lactate synergistic H + is secreted into the extracellular to reduce the pH of the tumor microenvironment, favoring tumor invasion and metastasis.^[^ ^5^ ^]^ Lactate in the tumor microenvironment can also serve as a proinflammatory and immunosuppressive molecule to affect tumor growth, which is closely related to the effect of immunotherapy.^[^ ^6^ ^]^ Recently, it has been found that lactate can be added as a modification molecule to the lysine residues of histone or other proteins, thereby affecting proteins biological function through participating in the gene expression regulation and the post‐translational modification.^[^ ^7^, ^8^ ^]^ Beyond its role as a metabolite, lactate also functions as an extracellular signaling molecule participating in the biological processes of tumor by activating HCAR1.^[^ ^9^, ^10^, ^11^ ^]^ HCAR1, also known as the G protein‐coupled receptor 81 (GPR81), depends on the specific activation of L‐lactate (EC50 ≈ 5 mm) in humans and mediates the development of multiple malignancies, such as breast cancer, pancreatic ductal adenocarcinoma, colorectal cancer, and lung cancer.^[^ ^12^, ^13^ ^]^

OS is also one of the classic “Warburg effect” cells, and this property has been reported as early as 2001 by Liu H et al.^[^ ^14^ ^]^ OS cells exhibit elevated lactate levels both intracellularly and extracellularly; however, the role of lactate as a signaling molecule in OS through HCAR1 activation remains to be fully elucidated. To this end, the present study was conducted to explore the presence of lactate/HCAR1 signaling in OS as well as its role and mechanism in the malignant biological behavior of OS. Through a series of experimental studies, it was found that lactate‐activated HCAR1 mediated the dephosphorylation of STAT1/2 via β‐Arrestin2/PP2A, thereby promoting OS progression. Overall, this finding expands the molecular mechanisms underlying the malignant behavior of OS and may facilitate the development of new targeted drugs for OS.

## 2 Results

### 2.1 HCAR1 Upregulation Predicting Poor Prognosis in OS

**Figure 1:**
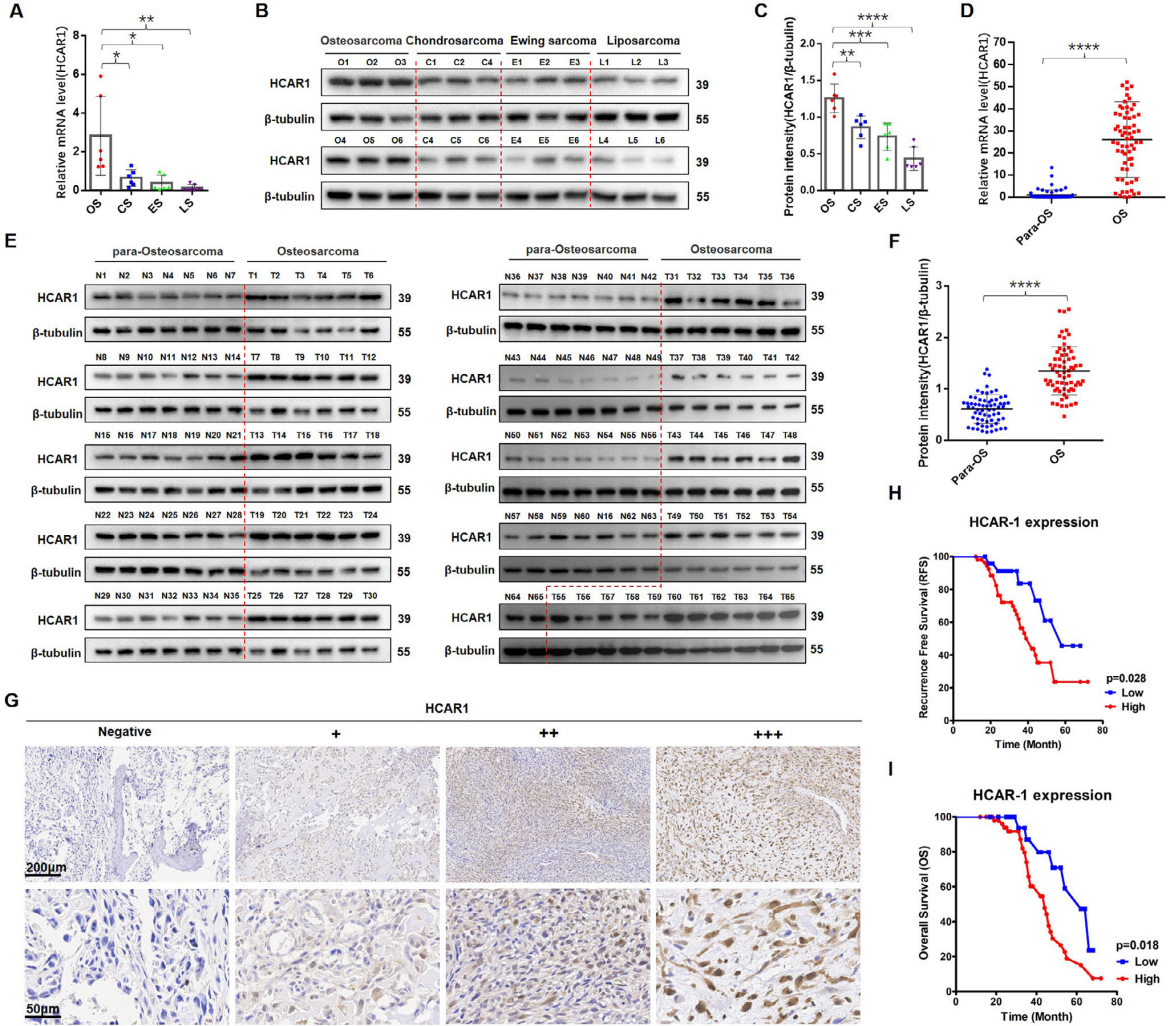
HCAR1 is significantly upregulated in OS and inversely correlated with poor prognosis. A–C) HCAR1 mRNA and protein levels in OS and other sarcoma tissues (including Chondrosarcoma, Ewing sarcoma, and Liposarcoma) by qRT‐PCR and Western blot. D–F) HCAR1 mRNA and protein levels in OS and adjacent tissues across 65 samples by qRT‐PCR and Western blot. G) Five‐micrometer (5‐µm) sections analyzed by IHC using anti‐HCAR1 antibodies. The quantitative analyses (Negative and + = low expression, + + and + + + = high expression) were performed for each sample (also see Figure S2, Supporting Information). H) Kaplan–Meier curve showing the correlation between HCAR1 expression levels and recurrence‐free survival rate (RFS) in OS patients. I) Kaplan–Meier curve showing the correlation between HCAR1 expression levels and overall survival (OS) in OS patients. Statistical analysis was performed using one‐way ANOVA (A,C) and unpaired *t*‐test (D,F). Error bars show means ± SD. *p < 0.05, **p < 0.01,***P < 0.001, and ****P < 0.0001. Scale bars, 200 or 50 µm.

First, the publicly available TCGA database of sarcoma data was used to analyze the HCAR family members correlated with survival. Kaplan–Meier curves showed that HCAR1 (GPR81) was inversely correlated with survival prognosis in sarcoma, while HCAR2 and HCAR3 showed no correlation across sarcoma samples (Figure S1, Supporting Information). Following that, the expression of HCAR1 in OS and other sarcoma tissues (including Chondrosarcoma, Ewing sarcoma, and Liposarcoma) was detected using qRT‐PCR and Western blot, and it was found that HCAR1 was significantly up‐regulated in OS tissues as compared to that in other sarcoma tissues (**Figure** **1**A–C). Subsequently, the expression of HCAR1 in OS and adjacent tissues across 65 samples from the hospital was examined using the same method, and the results suggested that HCAR1 was significantly up‐regulated in OS tissues as compared to that in adjacent tissues (Figure 1D–F). The in situ expression of HCAR1 protein by IHC staining was detected in 80 OS samples, and the quantitative analysis was performed for each sample (Figure S2, Supporting Information). According to the immunohistochemical scores of HCAR1, 80 OS samples were divided into high expression (+ + and + + +) and low expression (Negative and +) groups (Figure 1G). Elevated HCAR1 expression correlated with decreased progression‐free survival rate (PFS) and overall survival (OS) rate (Figure 1H,I) and an increased frequency of lung metastases (**Table** **1**, p **= 0.049**). All these findings demonstrated that HCAR1 was aberrantly upregulated in OS, and was inversely associated with the poor prognosis in patients.

**Table 1:** Associations between HCAR1 expression and the clinicopathological characteristics of OS.

Features	No.	HCAR‐1 expression		χ^2^	P
		High	Low		
**Gender**					
male	42	30	12	0.295	0.589
female	38	25	13		
**Age**					
≤20	48	31	17	0.97	0.461
>20	32	24	8		
**Histologic type**					
Osteoblastic	42	28	14	0.95	0.622
Osteolytic	10	6	4		
Mixed	28	21	7		
**Location**					
spine	54	38	16	0.203	0.797
extremities	26	17	9		
**Tumor size**					
Large (>5 cm)	48	36	12	2.16	0.15
Small (≤5 cm)	32	19	13		
**Enneking stage**					
I	16	8	8	7.023	**0.03**
II	28	17	11		
III	36	30	6		
**Surgical mode**					
en bloc	48	32	16	0.244	0.806
intralesional	32	23	9		
**Recurrence**					
Absence	46	27	19	5.093	**0.029**
Presence	34	28	6		
**Lung Metastasis**					
Yes	36	30	6	6.479	**0.015**
No	44	25	19		
**Last Status**					
Alive	42	24	18	5.545	**0.029**
Dead	38	31	7		

### 2.2 Lactate Acting on HCAR1 Promoting OS Cell Proliferation and Migration

**Figure 2:**
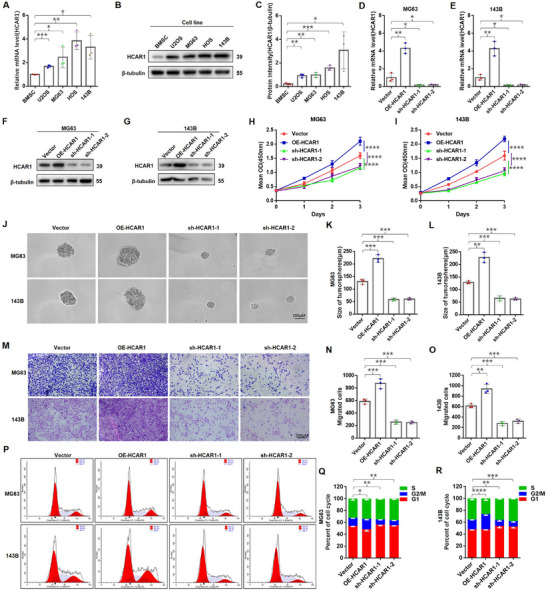
Lactate activating HCAR1 drives OS cell proliferation, migration, and invasion. A–C) HCAR1 mRNA and protein levels in BMSCs and OS cell lines by qRT‐PCR and Western blot. D–G) Validation of the mRNA and protein expression of HCAR1 gain and loss in MG63 and 143B cells by qRT‐PCR and western blotting assays (also see Figure S3F,G, Supporting Information). H,I) CCK8 assay and proliferation activity of the indicated OS cells featuring over‐expression or knockdown HCAR1. J–L) Soft agar clone formation assay as well as the size and number of the tumor sphere‐formation of the indicated OS cells, which over‐expression or knockdown HCAR1 (also see Figure S3H,I, Supporting Information). M–O) Transwell assays to evaluate the effects of HCAR1 on the migration and invasion of the indicated OS cells. Representative images and quantification of relative migrated cells are presented. P–R) Cell cycle was evaluated by the effects of HCAR1 function using flow cytometry in the indicated OS cells. Evaluated HCAR1 significantly promoted cell population at G2/M phase. Statistical analysis was performed using one‐way ANOVA (A,C–E,H,I,K,L,N,O,Q,R). Error bars show means ± SD. *P < 0.05, **P < 0.01, ***P < 0.001, and****P < 0.0001.

Metabolic reprogramming is crucial for cancer development,^[^ ^16^ ^]^ with OS likely arising from bone marrow mesenchymal stem cells (BMSCs) that preferentially engage in glycolysis.^[^ ^17^, ^18^, ^19^ ^]^ Therefore, the extracellular lactate concentration of BMSCs and OS cells was hereby initially detected, and it was found that the extracellular lactate concentration of OS was significantly higher than BMSCs, indicating the more active “Warburg effect” of OS cells (Figure S3A, Supporting Information). Meanwhile, HCAR1 expression was observed to be markedly higher in OS cells than in BMSCs (**Figure** **2**A–C). Additionally, OS cell cultures were supplemented with lactate, and CCK8 assays were conducted, revealing that lactate dose‐dependently increased OS cell proliferation. OS cell had the strongest activity at the extracellular lactate concentration of 10 mm (Figure S3B–E, Supporting Information).

To determine the function of HCAR1 in OS, HCAR1 in MG‐63 and 143‐B cells was stably overexpressed or knocked down via lentiviral infection, as the intervention efficiency was detected by qRT‐ PCR (Figure 2D,E) and Western blotting (Figure 2F,G; Figure S3F,G, Supporting Information). CCK8 assay, soft agar clone formation assay, and transwell assays showed that HCAR1 overexpression enhanced the proliferation, tumor spheres, migratory, and invasive capabilities of MG‐63 and 143‐B cells, whereas HCAR1 knockdown substantially reduced these properties in both cell lines (Figure 2H–O; Figure S3H,I, Supporting Information). Moreover, 10 mm lactate stimulation significantly promoted OS cells sphere‐formation capacity, migration, and invasion capacity, yet the above oncogenic effect of lactate was significantly attenuated after HCAR1 knockdown. Interestingly, giving lactate stimulation to HCAR1 overexpressing cells did not further enhance the above malignant behavior (Figure S3J–Q, Supporting Information), indicating this signaling effect in OS cells was not infinitely amplified by the increase of receptors or ligands.

Finally, flow cytometry was then used to determine whether cell cycle changes were induced when HCAR1 level was decreased or increased in MG‐63 and 143‐B. It was found that HCAR1 overexpression significantly increased the cell proportion in G2/M phase, while HCAR1 knockdown decreased the cell proportion in G2/M phase, suggesting that HCAR1 promoted cell cycle progression in OS (Figure 2P–R).

### 2.3 Lactate Activating HCAR1 Inhibiting the Transcriptional Activity of STAT1/2 in OS Cells

**Figure 3:**
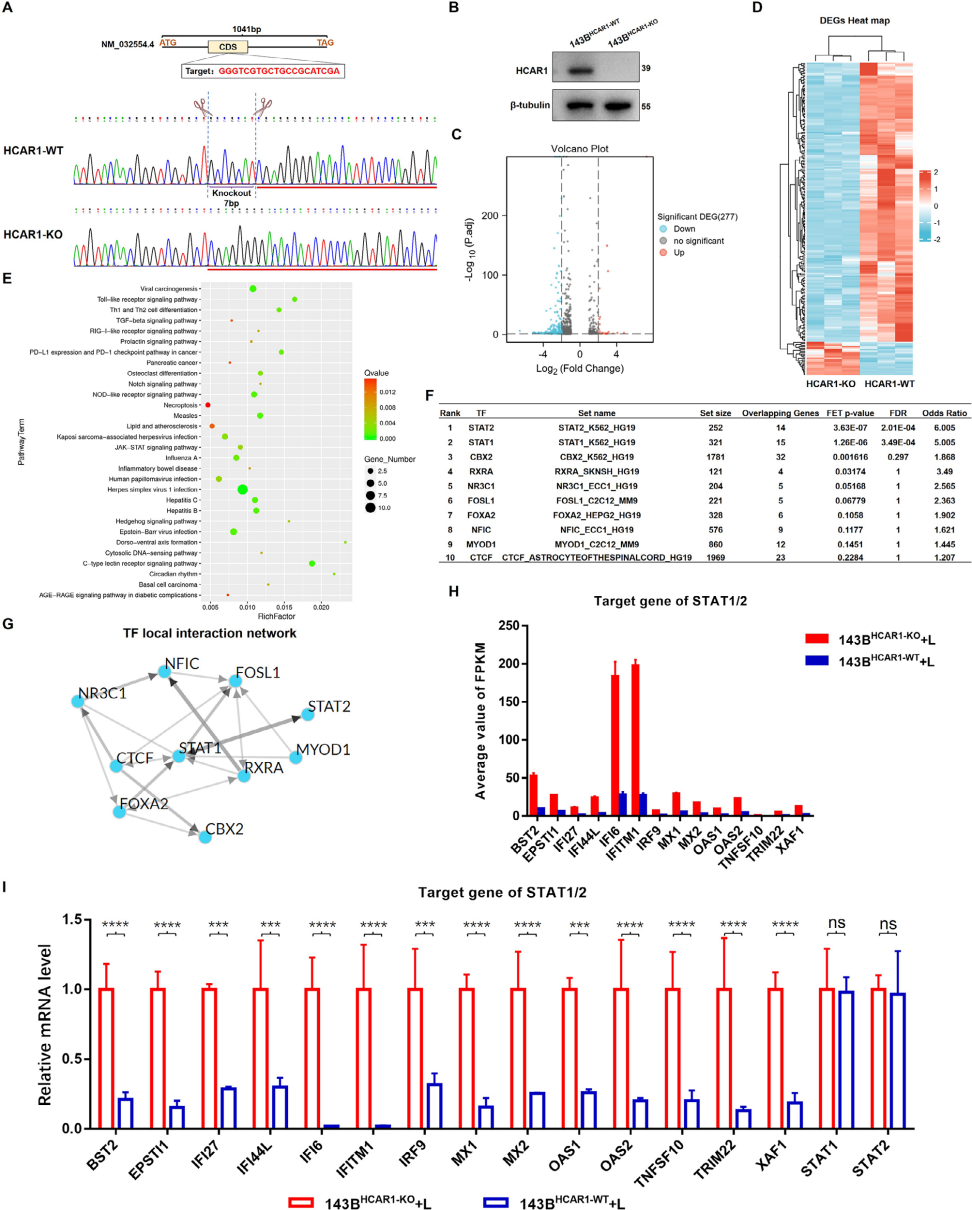
Lactate activating HCAR1 suppresses STAT1/2 transcriptional activity, thereby downregulating the expression of antioncogene downstream of STAT1/2. A) CRISPR‐Cas 9 performed to construct HCAR1‐knockout 143B cells. B) Validation of the knockout efficiency of HCAR1 in 143B cells by Western blot. C,D) RNA sequencing performed in wild‐type cells and HCAR1 knockout cells after lactate stimulation. Difference analysis of the transcriptome sequencing data was performed using the R Statistical Software. E) KEGG enrichment performed on the differential genes. F) ChIP‐X Enrichment Analysis 3 (ChEA 3) enriching the TFs for differential genes. G) Cytoscape visualizing co‐regulatory network of TFs. H) Differential analysis of the transcriptome sequencing data of STAT1/2 target genes. I) Validation of the effect of HCAR1 gain and loss on the mRNA levels of STAT1/2 and its target genes by qRT‐PCR. Statistical analysis was performed using one‐way ANOVA. (I). Error bars show means ± SD. ***P < 0.001, and ****P < 0.0001; NS, not significant.

To reveal the potential molecular mechanism, HCAR1 in 143B cells was knocked out by CRISPR‐Cas 9 (**Figure** **3**A,B). RNA sequencing was performed in wild‐type cells and HCAR1 knockout cells following lactate stimulation, and 277 genes with significantly up or down regulated were identified through differential analysis (Figure 3C,D). KEGG and GO enrichment suggested that these differential genes were mainly involved in regulating the transcription of RNA polymerase II promoter in nucleus, osteoclast differentiation, and JAK‐STAT signaling pathway (Figure 3E; Figure S4, Supporting Information). ChIP‐X enrichment analysis 3 was used for transcription factors (TFs) enrichment for 277 differential genes. Among the top 10 transcription factors obtained by screening, STAT 2 and STAT 1 were the two TFs with the most significant *p*‐value (Figure 3F). Moreover, in the visualized co‐regulatory network of these 10 TFs, STAT 1 was found to be located at the center of the network (Figure 3G). However, STAT 1 and STAT 2 were both overlapped and mutually regulated in function. Thus, it was speculated that STAT 1 and STAT 2 played a key role in lactate/HCAR1‐mediated transcriptome changes and malignant behavior of OS cells. Further analysis revealed that a total of 14 genes (including IFITM1, EPSTI1, TNFSF10, TRIM22, IFI27, BST2, IFI6, IFI44L, XAF1, OAS1, OAS2, IRF9, MX2, and MX1) are co‐regulated by both STAT1 and STAT2. With the exception of TRIM22, all other genes were confirmed as STAT1/2 target genes through ChIP‐PCR validation (Figure S5, Supporting Information). Moreover, upon lactate stimulation, the expression of all these genes was significantly downregulated in wild‐type cells (Figure 3H). Subsequently, further validation was performed by qRT‐PCR, and the results were completely consistent with the transcriptome data (Figure 3I). The above results indicated that the lactate activation of HCAR1 inhibited the transcriptional activity of STAT 1/2.

Based on the open‐access TARGET database, the correlation between the 14 genes and the survival prognosis of OS patients was also analyzed. The Kaplan–Meier curves showed that among the 14 differential genes, 5 were positively associated with the survival prognosis of OS (Figure S6, Supporting Information), the higher the expression, the better the prognosis, confirming their anti‐cancer effect in OS. Lactate activation of HCAR1 indeed suppressed STAT1/2 transcriptional activity and downregulated the expression of target genes, which might represent an intrinsic mechanism promoting tumor progression.

### 2.4 β‐Arrestin2 Mediating the Downregulation Signal of Lactate/HCAR1 on STAT1/2 Phosphorylation

STAT1/2 transcriptional activity is contingent upon tyrosine phosphorylation(STAT1 Y701 and STAT2 Y690). Upon cytokine stimulation, such as by interferon and transforming growth factor‐β, phosphorylated STAT1/2 dimers translocate to the nucleus to bind DNA and initiate transcription.^[^ ^20^, ^21^ ^]^ To determine the possible regulatory mechanism of lactate‐activated HCAR1 on STAT1/2, the Phosphorylated and global levels of STAT1/2 in 143B and MG63 cells stimulated with 10 mm lactate were examined through Western blot. The results showed that the OS cells normally expressing HCAR1 presented a significantly reduced phosphorylation level of STAT1 Y701 and STAT2 Y690 after lactate stimulation. However, the phosphorylation level of STAT1 Y701 and STAT2 Y690 had already been significantly increased at the basal state after HCAR1 was knocked out or knocked down, and was not affected by lactate stimulation (Figure S7A,B, Supporting Information). Additionally, the other phosphorylation sites were not affected by either lactate or HCAR1 (Figure S7C–F, Supporting Information). Following that, immunofluorescence was performed to detect the in situ phosphorylation of STAT1/2 in OS cells. Lactate stimulation caused a marked reduction in fluorescence intensity in OS cells expressing HCAR1, an effect not seen in HCAR1 knockout or knockdown cells, corroborating Western blot data (Figure S7G–J, Supporting Information). It was therefore concluded that in OS, the lactate activation HCAR1 inhibited the phosphorylation of STAT1/2.

**Figure 4:**
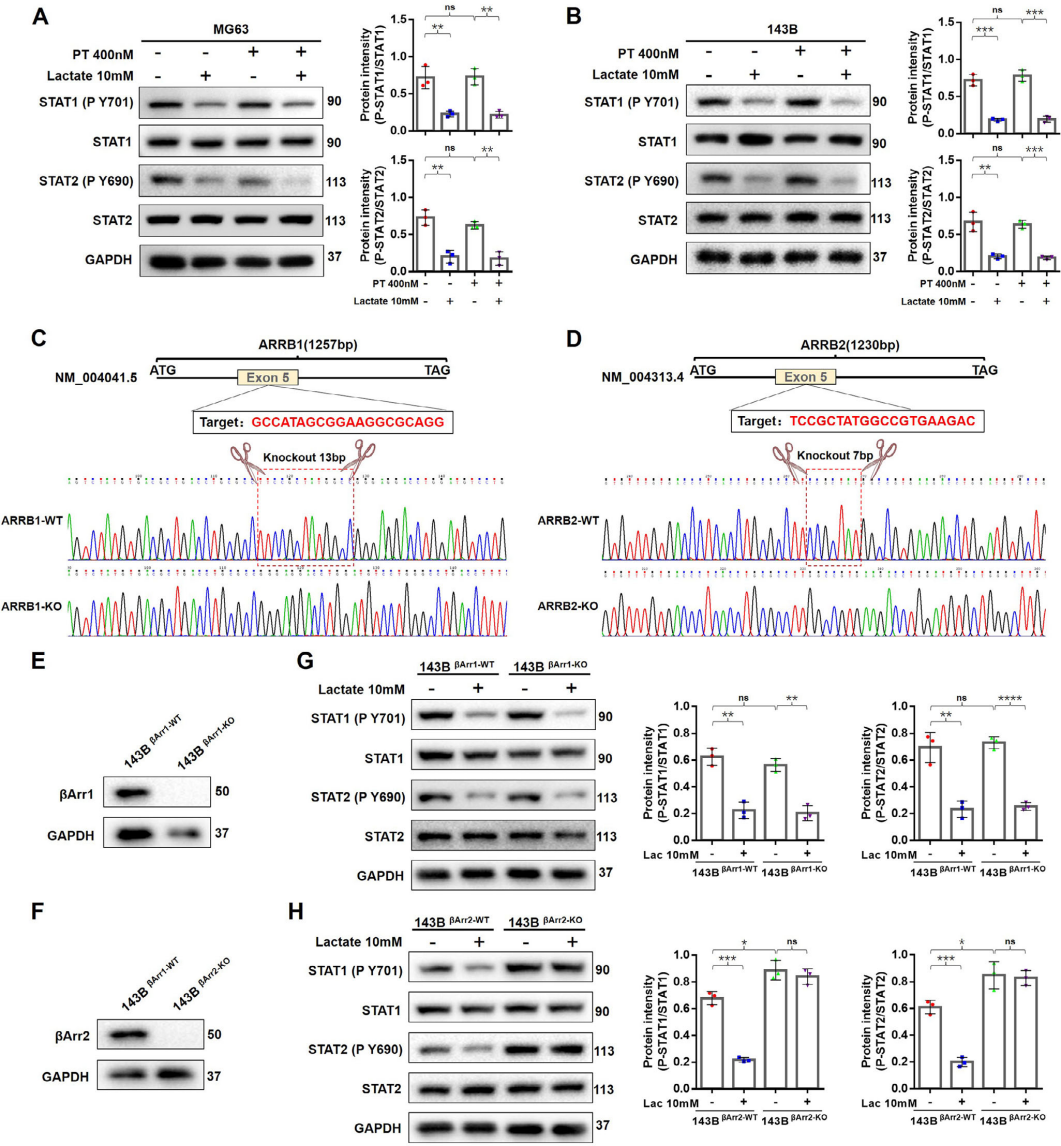
In OS cells, β‐Arrestin2 mediates the phosphorylation inhibition of STAT1/2 by lactate/HCAR1. A,B) The HCAR1/G_i/o_ signaling pathway of MG63 (A) and 143B (B) cells blocked by PT, and the phosphorylation level of STAT1/2 detected by Western blot. C,D) CRISPR‐Cas9 performed to knock out the 13 base pairs in β‐Arrestin1 and the 5 base pairs in β‐Arrestin2, and DNA sequencing detecting the knockout effect of β‐Arrestin1 (C) or β‐Arrestin2 (D) in 143B cells. E,F) Validation of the knockout effect of β‐Arrestin1(E) and β‐Arrestin2 (F) in 143B cells by Western blot. G,H) Western blot performed to evaluate the effect of β‐Arrestin1 knockout (G) or β‐Arrestin2 knockout (H) on the Phosphorylation levels of STAT1/2. Statistical analysis was performed using two‐way ANOVA test, followed by post hoc testing with Tukey's Honestly Significant Difference (HSD) test (A,B,G,H). Error bars show means ± SD. *P < 0.05, **P < 0.01, ***P < 0.001, and ****P < 0.0001; NS, not significant.

As a G protein‐coupled receptor, HCAR1 could perform signal transduction through Gi/o protein, the classical pathway,^[^ ^22^ ^]^ also through Arrestin, the non‐classical pathway.^[^ ^23^ ^]^ To determine the pathway that HCAR1 inhibits STAT1/2 phosphorylation, pertussis toxin (PT) was added while administering lactate stimulation, and the changes in STAT1/2 phosphorylation were subsequently detected. PT, which targeted Gi/o proteins to inhibit classical signaling,^[^ ^24^, ^25^, ^26^ ^]^ was used in MG63 and 143B cells. The results indicated that PT did not reverse STAT1/2 dephosphorylation, suggesting that HCAR1 might mediate its effects via a non‐classical pathway (**Figure** **4**A,B).

The ARRB1 gene encodes Arrestin2 (also known as β‐Arrestin1), while the ARRB2 gene encodes Arrestin3 (also known as β‐Arrestin2). These two molecules assume the function of mediating non‐classical signaling in peripheral tissues.^[^ ^27^ ^]^ Currently, there is no inhibitor of β‐Arrestin. Herein, 143B cells with β‐Arrestin1‐KO and β‐Arrestin2‐KO were constructed by CRISPR‐Cas9, and the knockout effect was verified by DNA sequencing and Western blot (Figure 4C–F). Following that, the phosphorylation level of STAT1/2 in β‐Arrestin1‐KO and β‐Arrestin2‐KO cells was examined. It was found that in β‐Arrestin1 knockout, the phosphorylation of STAT1/2 was still inhibited by lactate/HCAR1 signaling (Figure 4G). However, in β‐Arrestin2 knockout, the phosphorylation of STAT1/2 was already significantly increased in the basal state, and was not affected by lactate stimulation (Figure 4H). The findings indicated that β‐Arrestin2 mediated the inhibitory effect of lactate/HCAR1 on STAT1/2 phosphorylation. Consistent with previous reports, β‐Arrestin2 acted as a scaffold for kinases/phosphatases, transducing signals from GPCRs.^[^ ^28^ ^]^

### 2.5 Lactate Activating HCAR1 Enhancing PP2Aα Binding to STAT1/2 via β‐Arrestin2

**Figure 5:**
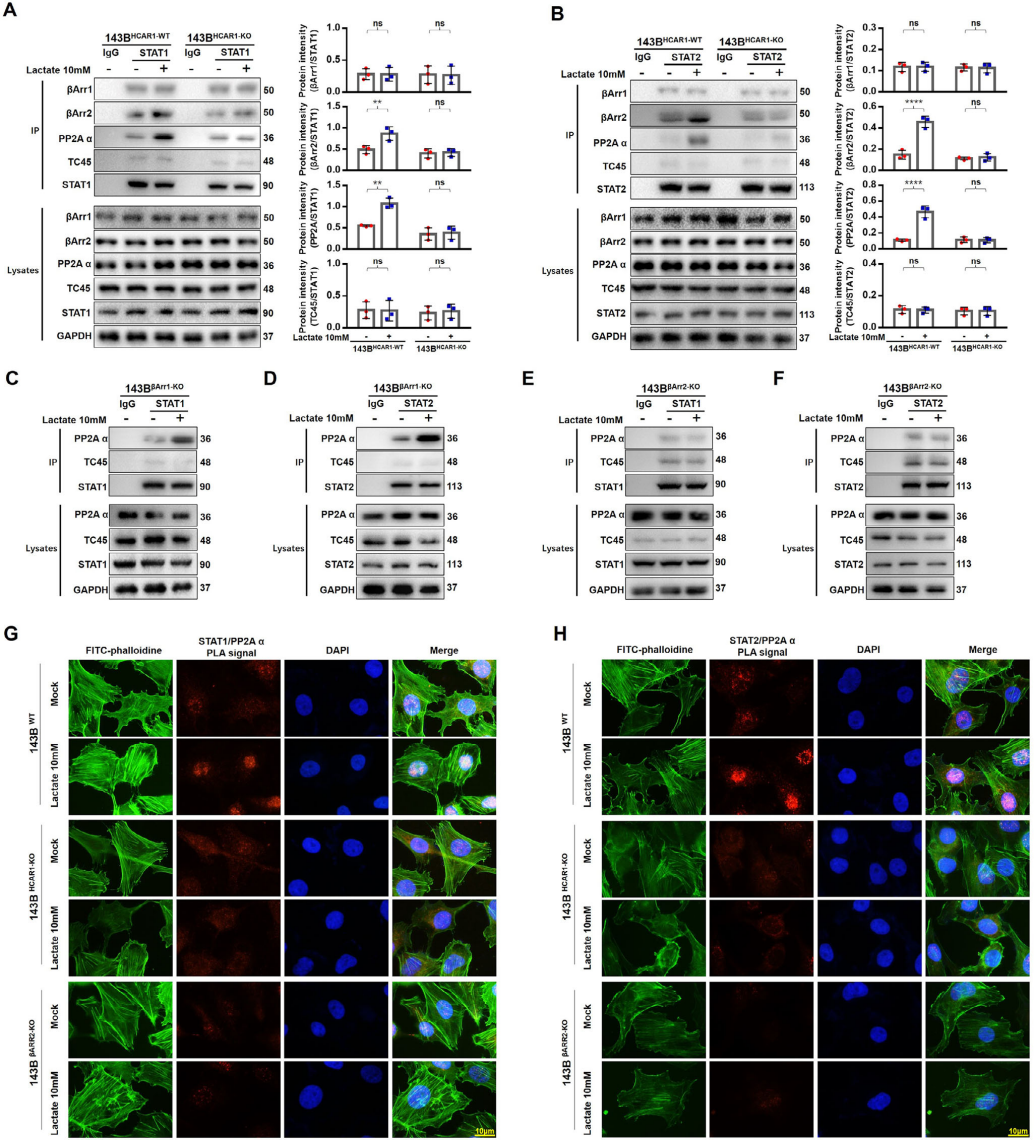
Lactate activating HCAR1 enhances PP2Aα binding to STAT1/2 via β‐Arrestin2. A,B) Immunoprecipitation experiments performed to assess the effect of lactate/ HCAR1 on the endogenous interaction of STAT1/2 and PP2Aα, β‐Arrestin1, β‐Arrestin2, PP2Aα, and TC45 of the indicated OS cells. 143B cell lysates were subjected to immunoprecipitation with anti‐STAT1/2, anti‐β‐Arrestin1, anti‐β‐Arrestin2, anti‐PP2Aα or anti‐TC45 antibody. The immunoprecipitates were then detected using the indicated antibodies. C–F) Immunoprecipitation experiments were performed to assess the effect of β‐Arrestin1 and β‐Arrestin2 on the endogenous interaction of STAT1/2 and PP2Aα, TC45 of the indicated OS cells. 143B cell lysates were subjected to immunoprecipitation with control IgG, anti‐STAT1/2, anti‐PP2Aα, or anti‐TC45 antibody. The immunoprecipitates were then detected using the indicated antibodies. G,H) Detection of the effect of lactate/HCAR1 on the relationship between STAT1/2 and PP2Aα in indicated OS cells by in situ PLA. PLA signal (Red fluorescence) appeared in situ of 143B cell. Statistical analysis was performed using two‐way ANOVA, followed by post hoc testing with Tukey's Honestly Significant Difference (HSD) test (A,B). Error bars show means ± SD. **P < 0.01, ****P < 0.0001; NS, not significant. Scale bars, 10 µm.

To further investigate the mechanism that lactate/HCAR1/β‐Arrestin2 mediated STAT1/2 dephosphorylation, 143B cells were used as target cells for lactate stimulation, and STAT1 was targeted for immunoprecipitation. The precipitated products were subjected to LC‐MS/MS analysis to identify proteins that might regulate STAT1/2 dephosphorylation (Figure S8A, Supporting Information). As a result, the proteins that specifically bound to STAT 1/2 were identified. Interestingly, among the top ten high‐abundance proteins, the dephosphorylation enzyme PP2Aα was present. (Figure S8B–D, Supporting Information). PP2A, a serine/threonine phosphatase with broad eukaryotic expression, exhibits tyrosine phosphatase activity under certain conditions.^[^ ^29^, ^30^ ^]^ Study have shown that β‐Arrestin2 can dephosphorylate AKT through its interaction with PP2A, where PP2A functions as a threonine phosphatase.^[^ ^31^ ^]^ However, what we have previously detected is the tyrosine phosphorylation level of STAT1/2; further focus will be placed on exploring which molecule is regulated by lactate/HCAR1/β‐Arrestin2 to mediate STAT1/2 dephosphorylation. Herein, immunoprecipitation experiments were designed, the bait protein STAT1/2 was precipitated with protein A/G magnetic beads, and the target proteins were detected using the indicated antibodies. The results showed that in wild‐type cells, lactate stimulation significantly increased the co‐precipitation of STAT1/2 with PP2Aα and β‐Arrestin2. While knocking out HCAR1, the co‐precipitation was not affected by lactate stimulation. Moreover, weak co‐precipitation of STAT1/2 with β‐Arrestin1 and tyrosine phosphatase receptor type C (TC45) occurred in either wild‐type or HCAR 1 knockout cells, and the precipitation amount was not affected by lactate stimulation (**Figure** **5**A,B; Figure S9, Supporting Information). Subsequently, STAT1/2 in β‐Arrestin1 or β‐Arrestin2 knockout cells was precipitated. It was found that when knocking out β‐Arrestin1, the lactate stimulation could still significantly enhance the co‐precipitation of STAT1/2 and PP2Aα, while knocking out β‐Arrestin2, the co‐precipitation of STAT1/2 with PP2Aα was decreased in the basal state, and was not affected by lactate stimulation (Figure 5C–F; Figure S10A–D, Supporting Information). The above results indicated that the lactate‐activating HCAR1 could promote PP2Aα‐STAT1/2 binding via β‐Arrestin2.

Subsequently, HCAR1 was stably overexpressed via lentiviral infection in 293T cells (Figure S10E, Supporting Information). The 293T cells overexpressing HCAR1 were transfected with fluorescent bimolecular complementary plasmids, VN173‐PP2Aα and VC155‐STAT1/2. The results demonstrated the presence of complementary fluorescence, while the fluorescence signal was enhanced after the addition of lactate stimulation (Figure S10F–H, Supporting Information). Furthermore, expressing exogenous STAT1/2‐Flag, PP2Aα‐HA, and β‐Arrestin2‐His in 293T cells also demonstrated that lactate stimulation significantly increased the co‐precipitation of the above‐mentioned molecules (Figure S10I,J, Supporting Information). Furthermore, we have generated truncated fragments of β‐Arrestin2, the mediator of the ternary complex assembly, and identified that STAT1/2 binds to the α‐helical region (Δ84–173) within the N‐domain of β‐Arrestin2, while PP2Aα interacts with its C‐terminus. Upon truncation βArr2Δ84–173, STAT1/2 failed to co‐precipitate with β‐Arrestin2, and co‐precipitation with PP2Aα was severely impaired. This demonstrates that β‐Arrestin2 plays an essential scaffolding role in facilitating the association between PP2Aα and STAT1/2 (Figure S11, Supporting Information).

For further validation, proximity ligation (PLA) was performed in 143B cell line. The results showed the presence of positivity as a red fluorescent signal, which indicated the in situ proximity between PPA2α and STAT1/2 in the nucleus of wild‐type 143B cells, and the red fluorescence signal could be significantly enhanced after lactate stimulation. However, either HCAR1 knockout or β‐Arrestin2 knockout resulted in a significant decrease of the fluorescence signal and was no longer regulated by lactate (Figure 5G,H). The activation of HCAR1 by lactate facilitated the binding of PPA2α to STAT1/2 via β‐Arrestin2, a non‐classical signaling molecule, as evidenced by the findings involving endogenous proteins, exogenous proteins, and OS cells. This interaction set the stage for the dephosphorylation of STAT1/2.

### 2.6 Inhibiting the Process of PP2A Dephosphorylating STAT1/2 Blocking the Pro‐Cancer Effects of Lactate/HCAR1/β‐Arrestin2

**Figure 6:**
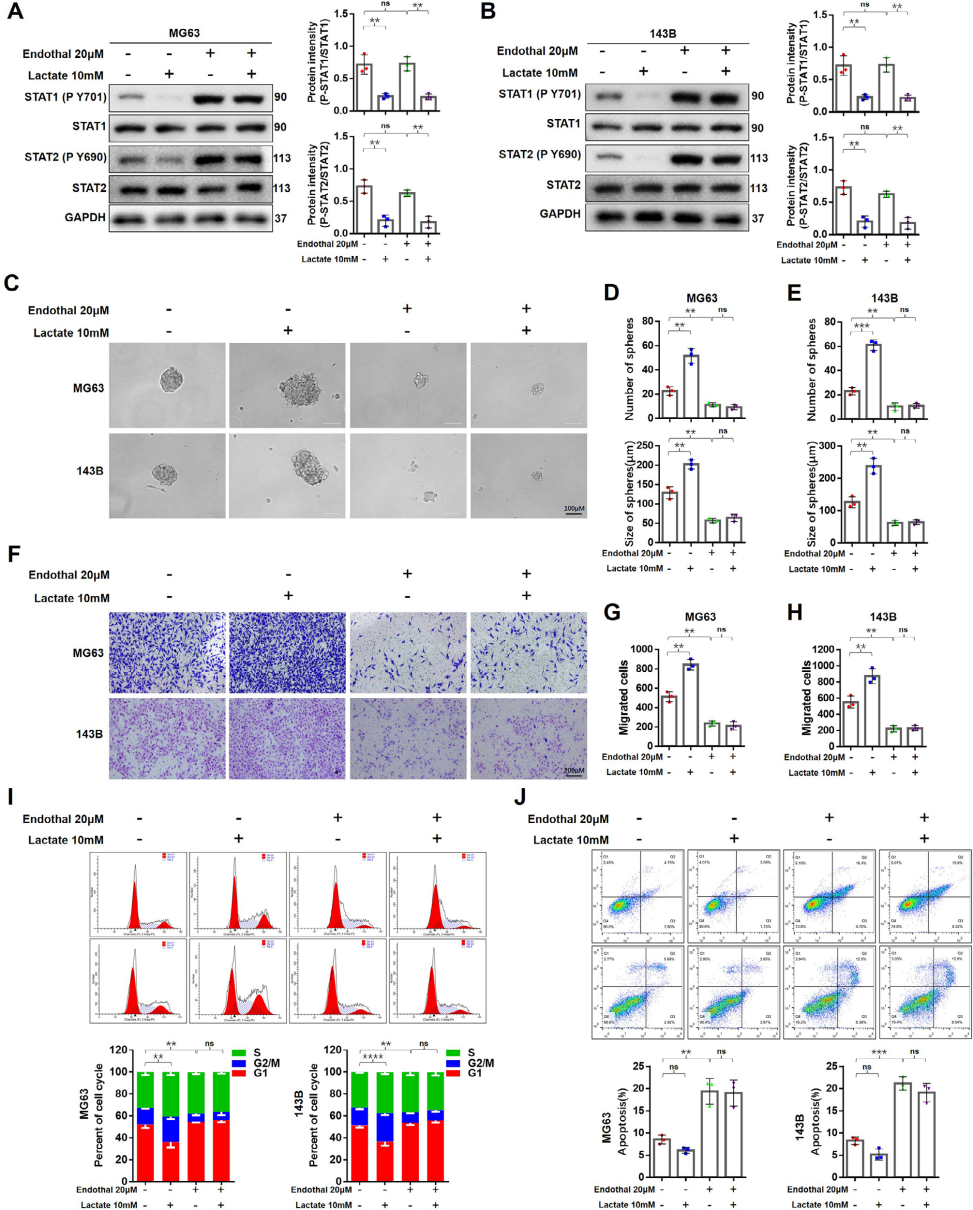
Inhibiting the process of PP2A dephosphorylating STAT1/2 could block the pro‐cancer effects of Lactate/HCAR1/β‐Arrestin2. A,B) MG63 and 143B cells treated with 10 mm lactate and 20 µM Endothall for 24 h, and the phosphorylation levels of STAT1/2 in the indicated OS cells measured by Western blot. C–E) Soft agar clone formation assay performed, as well as the size and number of the tumor sphere‐formation of the indicated OS cells treated with 10 mm lactate or 20 µm Endothall. F–H) Transwell assays performed to evaluate the effects of Endothall on the migration and invasion of the indicated OS cells. Representative images and quantification of relative migrated cells are presented. I,J) Cell cycle and apoptosis evaluated by the effects of Endothall intervention using flow cytometry in the indicated OS cells. Endothall significantly decreased the cell population at G2/M phase and increased the proportion of apoptotic cells. Statistical analysis was performed using two‐way ANOVA, followed by post hoc testing with Tukey's Honestly Significant Difference (HSD) test (A,B,D,E,G,H,I,J). Error bars show means ± SD. **P < 0.01, ***P < 0.001, and ****P < 0.0001; NS, not significant. Scale bars, 100 or 200 µm.

From the above experimental results, it was speculated that PP2A was the key for lactate/HCAR1/β‐Arrestin2 signaling to mediate STAT1/2 dephosphorylation. To confirm this speculation, Endothall, a PP2A selective inhibitor, was introduced.^[^ ^32^, ^33^ ^]^ As shown in **Figure** **6**A,B, lactate inhibited the phosphorylation of STAT1/2, and this inhibitory effect was completely reversed upon the addition of Endothall, demonstrating PP2A as the key enzyme in the dephosphorylation of STAT1/2 mediated by lactate/HCAR1/β‐Arrestin2. Furthermore, Endothall without altering STAT1/2 expression, counteracted lactate‐induced downregulation of TRIM22, IFI44L, OAS1, OAS2, and MX2, elevating their expression levels significantly above control levels (Figure S12, Supporting Information). These results indicated that Endothall could block the transcriptional activity inhibition of STAT1/2 mediated by lactate/HCAR1/β‐Arrestin2 pathway by targeting PP2A.

Subsequently, whether Endothall could block the pro‐cancer effects of lactate/HCAR1/ β‐Arrestin2 was further explored. MG63 and 143B cells were treated with lactate and Endothall, the number and volume of tumor sphere in both cell lines were significantly inhibited by Endothall, and the lactate stimulation had no effect on the inhibition (Figure 6C–E). Endothall also significantly inhibited cell migration, invasion, and cell cycle with or without lactate stimulation (Figure 6F–I). These results fully demonstrated that Endothall inhibited PP2A activity to upregulated STAT1/2 phosphorylation, which could block the pro‐cancer of lactate/HCAR1/β‐Arrestin2.

Furthermore, the results of apoptosis showed that Endothall significantly increased the proportion of apoptotic cells in OS and retained its pro‐apoptotic efficacy when co‐administered with lactate (Figure 6J), and demonstrating potential therapeutic significance.

### 2.7 HCAR1 Knockout or PP2A Inhibitor Suppressing OS Cell Proliferation and Metastasis

**Figure 7:**
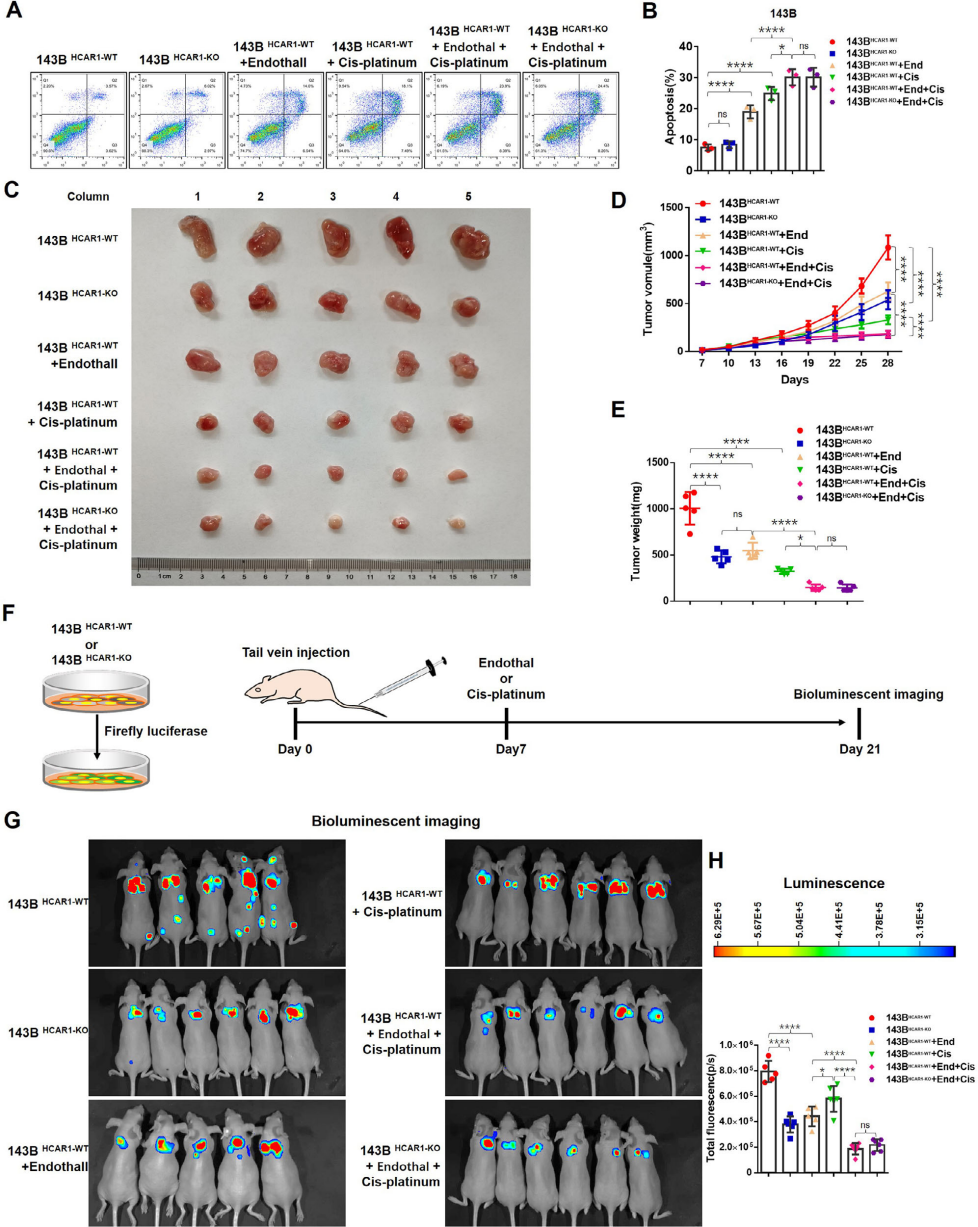
HCAR1 knockout or PP2A inhibitor promotes OS cell apoptosis in vitro and suppresses OS cell proliferation and metastasis in vivo. A,B) Using cisplatin as a reference (positive drug), the killing efficacy of HCAR1 knockout or Endothall on 143B cells evaluated by flow cytometry. C) Eight‐week‐old female BALB/c nude mice subjected to subcutaneous implantation into the dorsolateral side of the flank region for the indicated cell, and treated with Endothall, cisplatin, or a combination of both for two weeks on day 14. Representative images of the primary tumors from each group for twenty‐eight days are presented. D,E) Primary tumor volume measured every three days. Primary tumors were removed and weighed in each group. F) HCAR1 knockout 143B cells injected into nude mice through the tail vein, and the treatment of Endothall or cisplatin, or a combination of both performed on day 7. Bioluminescent imaging was performed on day 21. G,H) Bioluminescent imaging performed and total fluorescence computed to evaluate tumor growth and metastasis in the indicated tumor‐bearing mice. Statistical analysis was performed using multifactor ANOVA, followed by post hoc testing with Tukey's Honestly Significant Difference (HSD) test (B,D,E,H). Error bars show means ± SD. *P < 0.05, ****P < 0.0001; NS, not significant.

To further validate the lactate/HCAR1/β‐Arrestin2/PP2A signaling axis and its potential therapeutic significance for OS, the killing efficacy of HCAR1 knockout or Endothall on 143B cells was first evaluated by flow cytometry, using cisplatin as a reference. The results showed that Endothall's proapoptotic effect was lower than cisplatin; the combination of Endothall and cisplatin resulted in a significantly higher apoptotic rate than cisplatin alone. Moreover, the apoptosis rate caused by Endothall combining with cisplatin was not significantly different between wild‐type and HCAR1 knockout cells (**Figure** **7**A,B).

To delve into the role of HCAR1 in vivo, 143‐B cells with stable knockout of HCAR1 and control cells were implanted into the dorsolateral side of the flank region of BALB/c nude mice. The study revealed that HCAR1 knockout reduced tumor volume, slowed growth rate, and decreased tumor weight, effects comparable to those of Endothall‐treated wild‐type cells at the study's conclusion. The combination of Endothall and Cisplatin was highly synergistic in inhibiting tumor proliferation (Figure 7C–E). Ki67 staining intensity and cell count were directly proportional to tumor weight and volume within each group (Figure S13A, Supporting Information). Subsequently, the phosphorylation level of STAT1/2 in the tumor tissues was detected by Western Blot. The results revealed that either HCAR1 knockout or the addition of Endothall significantly increased the phosphorylation level of STAT1/2 in tumor tissues of mice. However, the group treated with cisplatin alone did not show the altered phosphorylation levels of STAT1/2, suggesting that cisplatin did not exert the anti‐cancer effect by affecting STAT1/2 (Figure S13B–D, Supporting Information).

Lung metastasis is the most common malignant behavior of OS. HCAR1 knockout and wild‐type 143B cells were injected into nude mice through the tail vein. The mice were treated with either cisplatin or Endothall after one week, and bio‐luminescent imaging was performed on day 21 (Figure 7F). The bio‐luminescent imaging showed that HCAR1 knockout or Endothall treatment could significantly inhibit the growth of pulmonary metastatic tumor, and its inhibitory effect was better than cisplatin. Moreover, it was also found that the combination of Endothall with cisplatin exhibited enhanced inhibition of lung metastasis compared to either drug alone (Figure 7G,H). In vivo experiments confirmed that blocking HCAR1/β‐Arrestin2/PP2A signaling axis suppressed tumor growth and metastasis, and augmented the therapeutic efficacy of cisplatin.

### 2.8 In OS Samples, HCAR1 Being Co‐Expressed with Ki67 yet Separated with Phosphorylated STAT1/2

**Figure 8:**
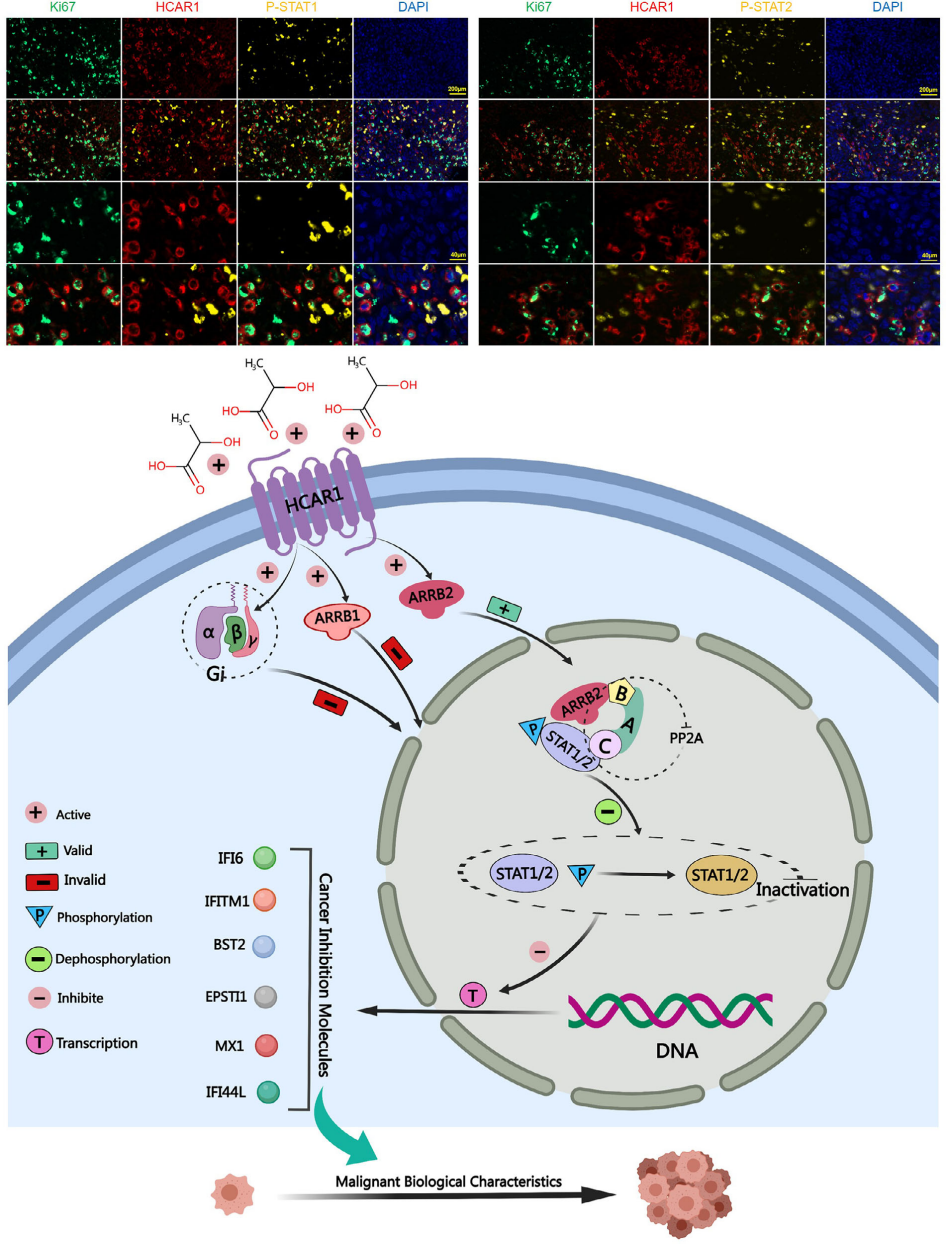
In OS clinical samples, HCAR1 is co‐expressed with Ki67 but separated with phosphorylated STAT1/2. The expression of HCAR1, Ki67, and phosphorylated STAT1/2 in OS clinical detected by immunohistochemistry. Scale bars, 40 or 200 µm. The function and mechanism of lactate‐activated HCAR1 mediating STAT1/2 dephosphorylation to promote OS development.

To validate the above findings in clinical samples, quadruple immunofluorescence (IF) was performed in OS samples to assess the relationship among HCAR1 expression, STAT1/2 phosphorylation, and cancer cell proliferation. It was found that HCAR1‐positive cells co‐expressed Ki67 and lacked or showed weak phosphorylation of STAT1/2. Conversely, HCAR1‐negative cells exhibited low or no Ki67 expression but had high levels of phosphorylated STAT1/2. In essence, in OS cells, HCAR1 expression is associated with Ki67 positivity and diminished or absent phosphorylated STAT1/2 (**Figure** **8**). The above study further confirmed that HCAR1 dephosphorylating STAT1/2 promoted OS malignant biological behavior in clinical samples. In conclusion, the study highlighted the function and mechanism by which lactate‐activated HCAR1 mediated STAT1/2 dephosphorylation to promote OS development, which could be weakened by HCAR1 knockout or PP2A inhibition (Figure 8).

## 3 Discussion

In order to find effective therapeutic targets and develop new therapeutic means, scholars worldwide are committed to exploring the molecular mechanism of OS. However, due to the high heterogeneity of OS cells and the strong environmental strain capacity, no substantial progress has been made. This study avoided the heterogeneous characteristics of OS and started from its commonality “Warburg effect” to explore the potential mechanism of lactate microenvironment on the malignant biological behavior of OS and provide some clues for the development of new therapeutic strategies.

The pro‐cancer effect of lactate has been gradually confirmed, which plays an indispensable role in OS progression. In humans, lactate functions as a signaling molecule to activate its specific receptor, HCAR1. Studies have also shown that lactate activation of HCAR1 has a pro‐cancer effect in breast cancer, lung cancer, colon cancer, cervical cancer, and glioblastoma.^[^ ^34^, ^35^, ^36^, ^37^, ^38^ ^]^ Based on sarcoma data from the TCGA database and our clinical cohort samples, we consistently observed that high HCAR1 expression was inversely correlated with tumor grade, stage, and survival rates in OS patients, albeit in a single‐center study with a limited sample size. In subsequent studies, it was found that overexpression of HCAR1 or giving additional lactate stimulation promoted the proliferation, invasion, and cell cycle of OS cells. Upon lactate stimulation following HCAR1 knockdown, the pro‐cancer effects were significantly diminished, suggesting a pro‐oncogenic role for lactate‐activated HCAR1 in OS development.

Using transcriptome sequencing and TFs enrichment analysis, it was found that the lactate activation of HCAR1 in OS resulted in the expression down‐regulation of some anti‐oncogenes regulated by both STAT1 and STAT2. On this basis, it was hereby speculated that the oncogenic properties of HCAR1 in OS might be associated with the transcriptional activity of STAT1/2. STAT1/2 are members of the signal transducer and activator of transcription (STAT) protein family. Once phosphorylated, STAT1/2 form homodimers or heterodimers, translocate to the nucleus, and bind to specific DNA regulatory elements to modulate target gene transcription.^[^ ^39^, ^40^ ^]^ The present results showed that STAT1/2 could mediate the transcription of 14 differential genes in OS cells, while according to TARGET database analysis, among the 14 differential genes, 5 were positively associated with the survival prognosis of OS. Previous studies also reported that IFI44L, TRIM22, OAS1, OAS2, and MX2 inhibited tumor proliferation and metastasis,^[^ ^41^, ^42^, ^43^, ^44^, ^45^, ^46^ ^]^ indicating that the transcriptional regulation of STAT1/2 could limit the development of OS. The lactate activation of HCAR1 was found to reduce the phosphorylation level of STAT1/2 and downregulate its transcriptional activity in OS cells, which might be the potential mechanism for the occurrence and development of OS.

HCAR1 signals through two main pathways: G protein‐mediated and β‐Arrestin‐mediated. To explore the downstream molecular mechanism of HCAR1 inhibiting STAT1/2 phosphorylation, PT was first used to block the Gi/o signaling pathway. The results showed that the phosphorylation of STAT1/2 was still inhibited by lactate, suggesting that HCAR1 might be signaling through β‐Arrestin pathway. There were no specific inhibitors for β‐Arrestin 1 and β‐Arrestin 2.β‐Arrestin 1 orβ‐Arrestin 2 was knocked out in OS cells using CRISPR‐Cas 9, and then the phosphorylation level of STAT1/2 was detected. It was found that β‐Arrestin 2 knocking out abolished the phosphorylation inhibition of HCAR1 on STAT1/2, suggesting that the lactate‐activated HCAR1 in OS inhibited STAT1/2 phosphorylation levels via β‐Arrestin 2 pathway.

Tyrosine dephosphorylation is a critical step in the inactivation of STATs within the nucleus. This process is primarily mediated by protein tyrosine phosphatases, which can act at the membrane‐associated receptor‐kinase complex or within the nucleus to dephosphorylate activated STAT dimers and recycle the latent STATs back to the cytoplasm.^[^ ^20^, ^21^ ^]^ TC45 is recognized as a major nuclear phosphatase responsible for STAT1 dephosphorylation. Wei Mo et al. proposed a model where β‐Arrestin 1 could function as a scaffold to recruit TC45 and promote nuclear STAT1 dephosphorylation.^[^ ^47^ ^]^

The present study revealed that PP2A interacted with STAT1/2 in OS cells, and the interaction was regulated by the lactate/HCAR1/ β‐Arrestin 2 pathway. This conclusion was based on the fact that lactate stimulation significantly increased PP2A‐STAT1/2 binding, resulting in STAT1/2 dephosphorylation, whereas knockout of either HCAR1 or β‐Arrestin 2 abolished this effect. Previous studies have reported that activated D2R or DRD5 could dephosphorylate IKK and AKT through β‐Arrestin2/PP2A signaling.^[^ ^31^, ^48^ ^]^ The phosphorylation sites of IKK and AKT are on serine/threonine residues, as PP2A is a serine/threonine phosphatase. However, under specific conditions, such as when bound to protein phosphatase 2 phosphatase activator, PP2A exhibits decreased serine/threonine phosphatase activity but increased tyrosine phosphatase activity.^[^ ^29^, ^30^ ^]^ According to the present study, the dephosphorylation sites of both STAT1 and STAT2 were all on tyrosine residues, and β‐Arrestin 2 functioned not only as a phosphatase scaffold, but also as a chaperone molecule of PP2A to upregulate the tyrosine phosphatase activity.

Numerous studies have reported that PP2A inhibits tumor progression through the phosphatase activity.^[^ ^49^, ^50^, ^51^ ^]^ For instance, PP2A can inhibit the proliferation and metastasis in lung cancer, breast cancer, gastric cancer, and other malignancies. However, this phosphatase can also activate several oncogenic pathways and positively participate in the oncogenic process. For instance, inhibition of PP2A radiosensitizes pancreatic cancers by modulating CDC25C/CDK1 and homologous recombination repair;^[^ ^52^ ^]^ PP2A dephosphorylates p38 enhances endothelial cell survival under stress condition.^[^ ^53^ ^]^ These findings demonstrate that the role of PP2A as either tumor‐suppressive or pro‐tumorigenic is not absolute but is context‐dependent, varying according to tumor type and the specific molecular targets it regulates. Our study showed that PP2A dephosphorylating STAT1/2 led to the expression downregulation of 5 differential genes, which was conducive to the proliferation and migration of OS cells, and played a pro‐cancer role. Therefore, PP2A was considered the key regulatable molecule in lactate/HCAR1/β‐Arrestin2 signaling axis. To further validate the proposed conclusion, cell and animal experiments were performed using Endothall, a small molecule inhibitor with high selectivity for PP2A. The results showed that Endothall almost completely abolished the promoting effects of lactate‐activated HCAR1 on OS proliferation, migration, and cell cycle, while promoting apoptosis. The subcutaneous tumor bearing experiment in nude mice further confirmed the anti‐cancer effect of Endothall, and the combination of Endothall and Cisplatin was highly synergistic in inhibiting tumor proliferation and promoting apoptosis. In the lung metastasis model, Endothall alone performed even better than Cisplatin. These experimental results fully demonstrated the important role of the lactate/HCAR1/β‐Arrestin2/PP2A signaling axis in OS malignant biological behavior.

## 4 Conclusion

The present study comprehensively assessed the role of lactate‐activated HCAR1 in OS progression by analysis of patients’ tissue samples, in vitro cell experiments, and in vivo animal experiments. Lactate accumulation and HCAR1 overexpression are the important characteristics of OS and are associated with a poor prognosis. In OS, lactate‐activated HCAR1 promotesβ‐Arrestin 2 recruits PP2A to the activated STAT1/2 dimers, mediating STAT1/2 dephosphorylation and inhibiting the transcription of downstream antioncogenes to promote OS progression. Knockout of HCAR1 or administration of PP2A inhibitors suppresses the malignant biological behaviors of OS. The study provides a completely new molecular mechanism of HCAR1 and extends the cognition of altered metabolism and lactate accumulation in OS.

## 5 Experimental Section

### Cell Lines and Cell Culture

HEK293T, MG63, 143B, HOS, and BMSCs cells were cultured in DMEM with 10% fetal bovine serum (Gibco 11765054) + 1% Penicillin/streptomycin (Gibco 10099141) in a 37 °C incubator with 5% CO2, and BMSCs cells were stimulated with M‐CSF (100 ng/mL) for 3 days. U2OS cells were cultured in McCoy's 5A with 10% FBS + 1% Penicillin/streptomycin at 37 °C with 5% CO2. All cell lines were authenticated by short tandem repeat profiling (Data S1, Supporting Information) and confirmed to be Mycoplasma‐free.

### Mice and Housing Conditions

Subcutaneous tumorigenesis experiments were conducted using female BALB/c nude mice aged 4 weeks. The experimental animal procedures were approved by the Changzheng Hospital Institutional Animal Care and Use Committee (No.2019SY116, June 16^th^, 2019), and all efforts were made to minimize animal suffering.

### Study Participant Details

A total of 80 primary or recurrent OS clinical tissue samples were obtained from OS patients who underwent Osteosarcoma radical correction and related palliative surgery at the Department of Orthopedic Surgery in Chinese PLA General Hospital or Shanghai Changzheng Hospital (Shanghai, China). Each sample included osteosarcoma lesion tissue and paired normal non‐tumor tissue at least 5 cm away from the carcinoma focus for control. The inclusion criteria were patients: 1) who underwent radical correction in OS or related palliative surgery; 2) whose OS diagnosis was confirmed by pathology; 3) who had complete clinical data, pathological results, and follow‐up data; and 4) whose surgical pathological sections were well‐preserved. Freshly resected samples were stored at −80 °C or paraffin‐embedded. All procedures were conducted in accordance with the Declaration of Helsinki and approved by the Institutional Ethics Review Board of the said hospital (No. 2022SY086, Augest 21st, 2022). Written informed consent was obtained from all patients.

### Plasmid Construction

HCAR1 was PCR‐amplified from cDNA and cloned into the pLVX‐IRES‐Puro vector for lentiviral packaging. shRNA oligos were designed by Sigma‐Aldrich and synthesized by Sangon Biotech, then annealed and cloned into the pLVX‐KO‐IRES‐Puro vector for lentiviral packaging. PPP2CA was PCR‐amplified from cDNA and cloned into the pCE‐BIFG‐VN173 vector. STAT1 and STAT2 were PCR‐amplified from cDNA and Recombinant into the pCE‐BIFG‐VC155 vector. A Flag‐tag was incorporated at the N‐terminus of STAT1 and STAT2, a His‐tag at the N‐terminus of ARRB2, and an HA‐tag at the N‐terminus of PPP2CA. These tagged genes were then recombined into the pcDNA3.1+ vector via homologous recombination. Plasmid constructions were performed using the ClonExpress Ultra One Step Cloning Kit (Vazyme C115‐01). Primers, shRNA, and sgRNA sequences are provided in Table S1 (Supporting Information).

### Lentivirus Packaging and Infection

To generate stable cell lines expressing specific HCAR1 or shRNAs, HEK293T cells were hereby transfected with the appropriate lentiviral vector (pLVX‐IRES‐Puro‐HCAR1 or pLVX‐KD‐IRES‐Puro‐sh‐HCAR1‐1/2) and packaging plasmids (psPAX2 and pMD2.G) using Lipofectamine 3000 transfection reagent. Each virus‐containing supernatant was collected 48 h upon transfection and infected with the target cells (MG63 and 143B) at 70% confluence. Besides, 1 µg mL^−1^ puromycin was employed for drug‐based selection for one week.

### CRISPR/Cas9‐Mediated Genome Editing

The CRISPR/Cas9 technology was adopted to construct genomic HCAR1, ARRB1, and ARRB2 knockout in 143B cells. CRISPR/Cas9 editing was performed as described previously.^[^ ^15^ ^]^ Monoclonal 143B cells were then expanded for DNA extraction. Genomic regions flanking the sgRNA target sites were amplified by PCR and subjected to Sanger sequencing. Western blot analysis was subsequently used to verify the deletion of the target genes. The sequences of sgRNAs and primer for PCR used in the current study are listed in Table S1 (Supporting Information).

### RNA Isolation and Quantitative Reverse Transcriptase PCR

RNA was extracted from treated cells using TRIzol reagent (Invitrogen 15596‐026) and reverse transcribed to cDNA using a PrimeScript RT Reagent Kit with gDNA Eraser (TaKaRa RR037A) following the manufacturer's recommended protocols. Primers were designed from information available on the PrimerBank website. Quantitative reverse transcriptase PCR (qRT‐PCR) was performed using SYBR Premix Ex Taq (TaKaRa RR420A) in an ABI 7900HT Fast Real‐Time PCR System (Applied Biosystems). The gene‐specific primers used are listed in Table S1 (Supporting Information).

### Western Blot

Proteins were extracted by RIPA containing the phosphatase inhibitor and protease inhibitor. The protein concentration was determined by BCA kit (Beyotime P0012). 10–12% polyacrylamide gels were used to separate protein. Subsequently, the protein was transferred to PVDF membranes. After blocking in 5% BSA, the membranes were incubated with the indicated primary antibodies at 4 °C overnight, followed by the addition of the corresponding HRP conjugated secondary antibodies to the membranes.

### Immunohistochemical Staining

IHC staining was performed as described previously, using anti‐HCAR1 primary antibodies at a dilution of 1:100. The stained slides were scanned utilizing Pannoramic DESK and Pannoramic Scanner (3D HISTECH, Hungary). The staining intensity and area were recorded for each sample, followed by quantitative analysis using IHC Profiler, with scoring defined as follows: 0 = Negative, 1+ = Low Positive, 2+ = Positive, and 3+ = High Positive. Low and high expressions were defined according to the median immunoreactivity score.

### Measurement of Lactate

To measure the lactate‐producing capacity of different cells, cell culture supernatants were collected and diluted with distilled water to the appropriate fold‐ratio. Proteins were precipitated by centrifugation at 4 °C and 12 000 g for 10 minutes using a 10 kD spin column (ab93349). The supernatant was then collected, and lactate concentrations were determined using an l‐Lactate Assay Kit (Abcam, ab65330) according to the manufacturer's protocol.

### CCK8 Assay

Cells were seeded in a 96‐well plate at a density of 5000 cells per well. After 24‐h incubation, the cells were treated with the indicated agents for 24 h, and then 10 µL Cell Counting Kit‐8 (CCK‐8) solution was added to each well for 2 to 4 h (HY‐K0301, MedChemExpress, China). The optical absorbance (AD) at 450 nm was measured using an ELx800 microplate reader (BioTek Instruments Inc., USA).

### Soft Agar Colony Formation

Cell lines were harvested to obtain a density of 5 × 10 ^3^ cells/well. Subsequently, 1% agar was melted in a microwave and cooled to 40 °C in a water bath, and 2X DMEM was warmed to 40 °C in a water bath and maintained for at least 30 min to allow the temperature to equilibrate. Equal volumes of the two solutions were mixed to obtain 0.5% agar + 2X DMEM, and 0.6 mL of this solution was then added to each well and allowed to solidify. Subsequently, 0.7% agar was melted in a microwave and cooled to 40 °C in a water bath, and 2X DMEM was also warmed to 40 °C in a water bath. For plating, 3 mL of 2X DMEM and 3 mL of 0.7% agar were combined in a tube and gently mixed. Then, 0.6 mL of the resulting mixture was added to each replicate plate. The plates were incubated at 37 °C in a humidified incubator for 10–14 days, after which colonies were counted using a dissecting microscope.

### Transwell Assay

Migration and invasion assays were performed using transwell chambers in the presence of Matrigel (Corning 354480). Briefly, experimental cells (1 × 10^5^ cells/well) were seeded into the upper chamber with serum‐free medium, and 600 µL of 20% FBS was added to the lower chamber of the 24‐well plate. Following 24 h of incubation, the cells on the upper surface of the filter were completely removed by wiping them with a cotton swab. Then, the filters were fixed with 4% paraformaldehyde and stained with crystal violet (Beyotime C0121). The cells in 5 randomly selected visual fields were counted using a microscope (DM500; Leica, Wetzlar, Germany) at 100× magnification.

### Flow Cytometry

Cell‐cycle analysis was performed by propidium iodide (PI; Sigma‐Aldrich 537060) staining to quantify the sub‐G1, S, and G2 populations, which reflected the extent of cell cycle. Briefly, 1 × 10^5^ cells were seeded in a 6‐well plate. After 24 h, cells were treated with the indicated agents for 72 h, fixed with 70% ethanol, and stained with PI (50 µg mL^−1^). The stained cells were tested using SP6800 Spectral Cell Analyzer (Sony) and analyzed using the FlowJo software. For apoptosis assay, cells were treated with the indicated compounds and stained with 5 µL FITC‐Annexin V (BD Biosciences 556419) and 5ul PI (BD Biosciences 556463). After 15 min, the stained cells were analyzed using flow cytometry.

### RNA‐Seq

RNA isolation, library construction, and sequencing were performed on a BGISEQ‐500 (Beijing Genomic Institution, BGI). Gene expression analysis entailed calculating and normalizing reads to FPKM, followed by determining fold changes and applying a 2‐fold change cutoff to identify genes with significant expression alterations. GO and KEGG enrichment analyses were performed using the R package, taking significantly differentially expressed genes (FDR % 0.001) as target genes. ChIP‐X enrichment analysis 3 (ChEA 3) was utilized to enrich the transcription factors of differential genes, and a co‐regulatory visualization network of transcription factors was constructed using Cytoscape. Raw data files and processed files were uploaded to Bioproject database (PRJNA1184966). The RNA‐seq dataset are available at https://www.ncbi.nlm.nih.gov/bioproject/PRJNA1184966.

### Immunofluorescence

Cells were fixed with paraformaldehyde for 15 min and permeabilized with Triton X‐100 for 20 min at room temperature. Cells or tissue sections were blocked with 1% BSA solution for 1 h at room temperature and incubated with primary antibody (1:50–100) at 4 °C overnight. The secondary antibody (1:400) was diluted in PBS and incubated at room temperature for 1 h. The slides were counterstained with DAPI. Finally, cell nuclei were stained with DAPI (Servicebio G1012‐10ML), and the fluorescence signal was captured using a Leica TCS SP8 laser‐scanning confocal microscopy.

### LC‐MS/MS Assay

143B cells were harvested following a 3‐hour lactate stimulation. Total cell protein was extracted using NP‐40 Lysis Buffer supplemented with the protease inhibitor and immunoprecipitated with Anti‐STAT1. The immunoprecipitates were washed three times with PBST Buffer and separated by SDS‐PAGE. The bands of IgG group or STAT1 group were cut from the gel. The sliced gel samples were analyzed by liquid chromatography tandem mass spectrometry (LC‐MS/MS) performed by Shanghai Applied Protein Technology (APTBIO, China).

### Immunoprecipitation

For the immunoprecipitation of both exogenously expressed and endogenous proteins, cells were lysed in NP‐40 Lysis Buffer with a protease inhibitor cocktail. The lysates were incubated with primary antibodies or control IgG overnight at 4 °C in a rotating incubator, followed by a 4‐hour incubation with protein A/G magnetic beads (Bimake B23201) at 4 °C. The immunoprecipitates were washed three times with PBST Buffer and analyzed by immunoblotting.

### Proximity Ligation Assay

The cells were seeded onto slides, allowed to attach overnight, fixed with 4% paraformaldehyde, and permeabilized with 0.3% Triton X‐100 in PBS. The cells were processed using the manufacturers' protocol of the Duolink In Situ Red Starter Kit (Sigma‐Aldrich, DUO92101), where the cells were probed for either rabbit anti‐STAT1 or mouse anti‐PPP2CA or both, followed by ligation and amplification process. Finally, cell nuclei were stained with DAPI, cytoskeleton was stained with FITC‐Phalloidin, and the PLA signal was captured using the fluorescence microscope (IX71; Olympus Corporation, Tokyo, Japan).

### Bimolecular Fluorescence Complementation

293T cells were seeded into 24‐well plate, and transfected with pCE‐BIFG‐VN173‐PPP2CA or pCE‐BIFG‐VC155—STAT1/2 or both using Lipofectamine 3000 transfection reagent at 70% confluence. Subsequently, after 24 h of transfection, cells were stimulated with lactate for 3 h, and BiFC signals were captured using the fluorescence microscope (IX71; Olympus Corporation, Tokyo, Japan).

### Xenograft Tumor Assays

For the subcutaneous xenograft assay, female BALB/c nude mice aged 4 weeks were subjected to subcutaneous implantation into the dorsolateral side of the flank region for the 143B cells (5 × 10^6/^100 µL PBS). Intraperitoneal treatment with specific drugs (cisplatin: 5 mg/kg/2d, Endothall: 3 mg/kg/2d) was conducted for 6 cycles. Tumor size was measured every three days using a vernier caliper, and mean tumor volume was calculated using the following equation: volume = 0.52 × length × width. At study termination, xenograft tumors were excised, measured, and weighed after euthanasia.

### Bioluminescent Imaging

In vivo distant metastasis was monitored using bioluminescent imaging. Luciferase‐expressing 143B cells, established via lentiviral transduction, were tail‐vein injected into mice. After one week, the mice were treated with cisplatin or Endothall every other day for 2 weeks. Anesthetized mice were administered D‐luciferin potassium salt (AOK Chem, Shanghai) at 150 mg/kg intraperitoneally. Ten minutes later, they were imaged using the IVIS Imaging System with a 2‐minute exposure. Bioluminescence was measured, and photon counts per second were quantified with software (Living Image 3.2, Caliper).

### Chromatin Immunoprecipitation Followed by Polymerase Chain Reaction

Cells (typically 1 × 10^6^ to 5 × 10^6^ per IP) were crosslinked with 1% formaldehyde for 8–12 minutes at room temperature, quenched with glycine, lysed, and chromatin was sonicated to shear DNA into 200–1000 bp fragments. The soluble chromatin was pre‐cleared (optional) with Protein A/G beads, diluted, and incubated overnight at 4 °C with a specific antibody against the target protein (experimental) or non‐specific IgG (control); a portion was saved as Input DNA. Antibody‐chromatin complexes were captured with Protein A/G beads for 2–4 hours, then washed sequentially with low salt, high salt, LiCl, and TE buffers. Bound complexes were eluted with SDS/NaHCO_3_ buffer, and crosslinks were reversed by overnight incubation at 65 °C with NaCl. DNA was purified from the eluates and Input samples. Specific genomic regions were amplified from the purified DNA using PCR with target‐specific primers and standard thermocycling, with enrichment calculated relative to controls and Input.

### Statistical Analysis

All statistical analyses were performed using GraphPad Prism 7.0. Bioinformatics analysis was performed with R (version 4.2.1). Survival curves were plotted using the Kaplan‐Meier method and compared using log‐rank tests, with other data presented as the mean ± standard deviation (SD). Differences between groups were analyzed by Student's t‐test or ANOVA, assuming double‐sided independent variance. For evaluating differences between groups, the Fisher's exact test or Pearson's chi‐square test was carried out for categorical variables assuming double‐sided independent variance. A *p*‐value < 0.05 was regarded as statistically significant. ** indicates *P* < 0.01, *** indicates *P* < 0.001, and **** indicates *P* < 0.0001.

## Conflict of Interest

The authors declare no conflict of interest.

## Author Contributions

Z.H., Q.J., G.B., and Z.L. contributed equally to this work. L.T.L., W.T., H.Z.T., L.W.B., and M.Y. conceptualized and supervised the study. H.Z.T., J.Q., B.G.J., L.Z., Z.J., and W.Z.H. designed the methodology and performed validation. Z.J., C.J.S., X.B.Q., L.Y.G., S.J.Y., W.L.N., H.X., Z.C.L., T.K., D.F.L., and C.S.Y. performed data curation (provided animals, acquired and managed patients, provided facilities, etc.). H.Z.T., J.Q., B.G.J., L.Z., Z.Z.H., Z.Y.J., and Z.W.N. performed formal analysis and visualization (e.g., statistical analysis, biostatistics, computational analysis). H.Z.T., J.Q., B.G.J., L.Z., and C.G.H. wrote the original draft and reviewed and edited the manuscript. C.Y.M. and Y.X.H. gave administrative, technical, or material support (i.e., reporting or organizing data, constructing databases).

## Supporting information



Supporting Information

Supporting Information

Supporting Information

Supporting Information

Supporting Information

Supporting Information

Supporting Information

## Data Availability

The data that support the findings of this study are available from the corresponding author upon reasonable request.
