# Supplementary material for: Lactate Activates the HCAR1/β‐Arrestin2/PP2A Signaling Axis to Mediate STAT1/2 Dephosphorylation and Drive Osteosarcoma Progression
Source: Adv Sci (Weinh). 2025 Sep 16;12(45):e06214. doi: 10.1002/advs.202506214 (PMC12677626; doi:10.1002/advs.202506214)
Supplement: Supplementary file 1 — Supporting Information [file ADVS-12-e06214-s007.docx]

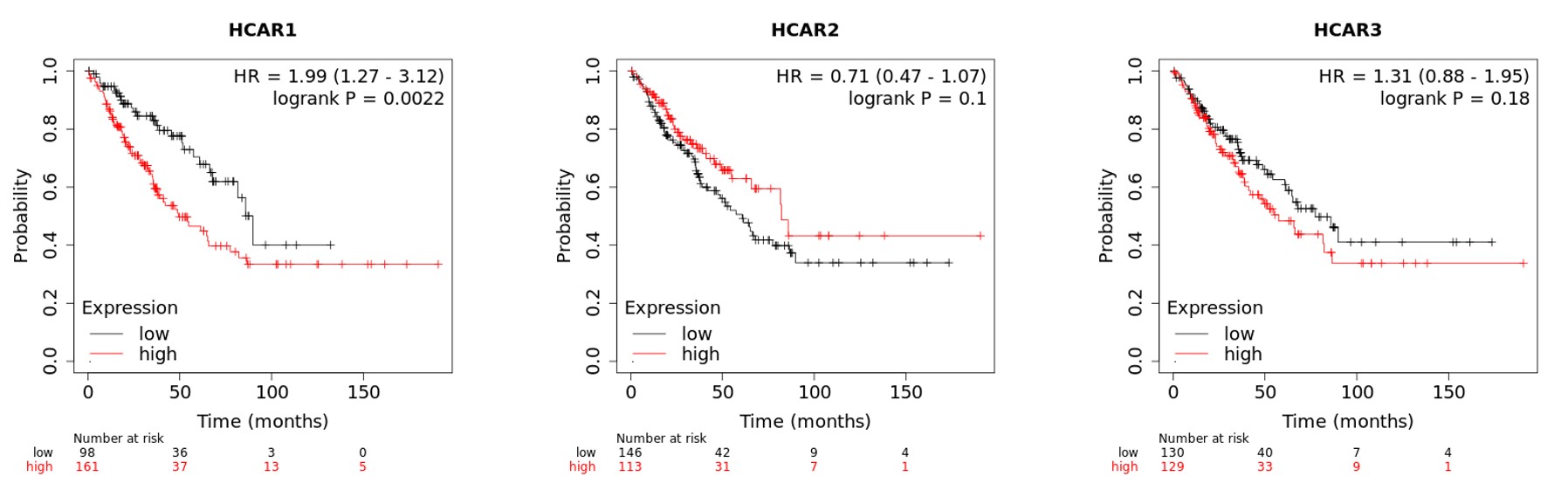


**S Figure. 1** The correlation between HCAR family members and sarcoma survival. Kaplan-Meier curve of sarcoma tissues, showing that higher HCAR1 expression was associated with poor prognoses based on TCGA database, while HCAR2 and HCAR3 showed no correlation across sarcoma samples.


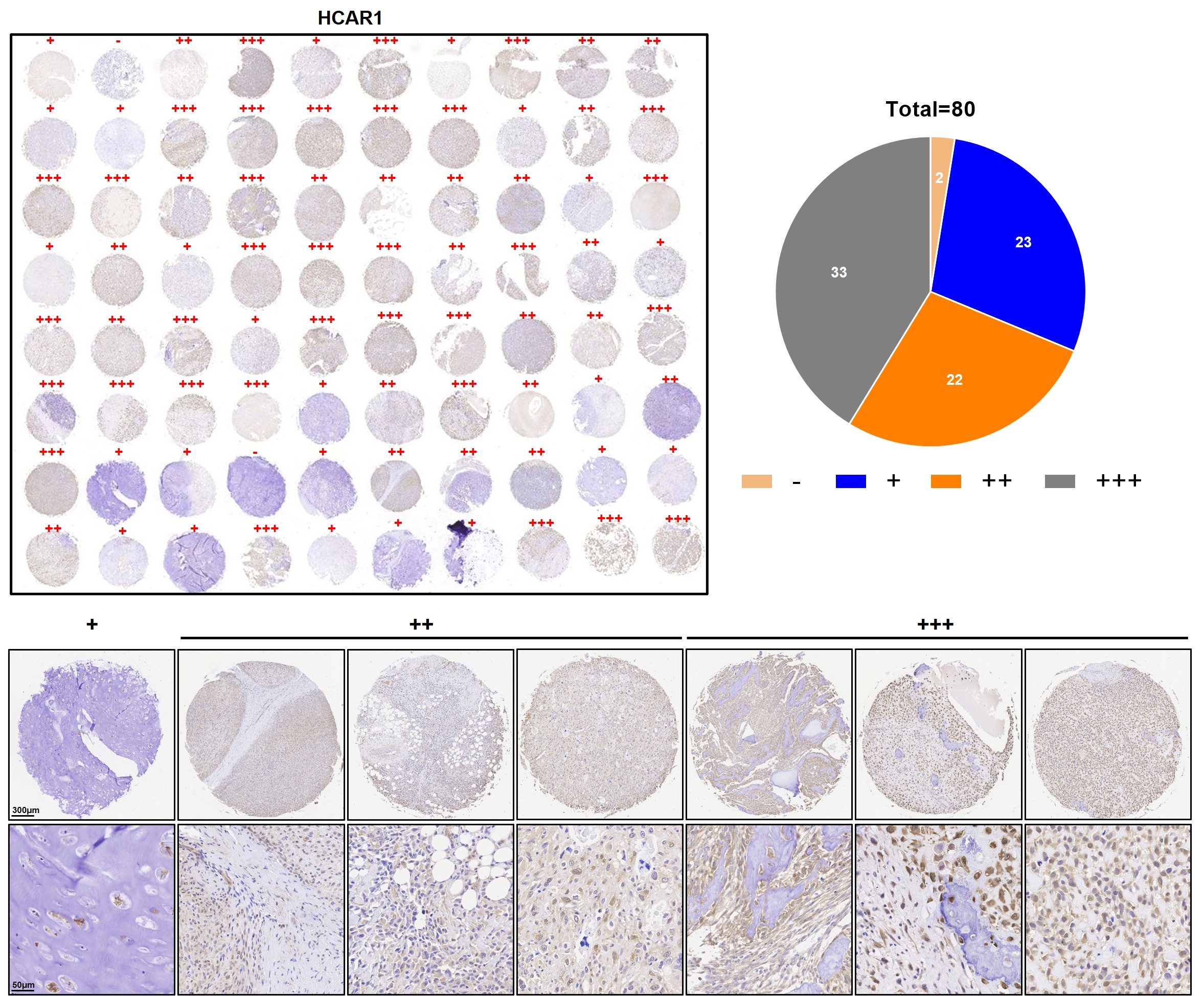


**S Figure. 2** The IHC staining of OS samples. Tissue microarray of 80 clinical samples cut to five-micrometer (5-µm) sections and analyzed by IHC using anti-HCAR1 antibodies. Based on the staining intensity and stained cells amount of HCAR1, the expression of HCAR1 was divided into four classes, namely, -, +, ++, and +++.


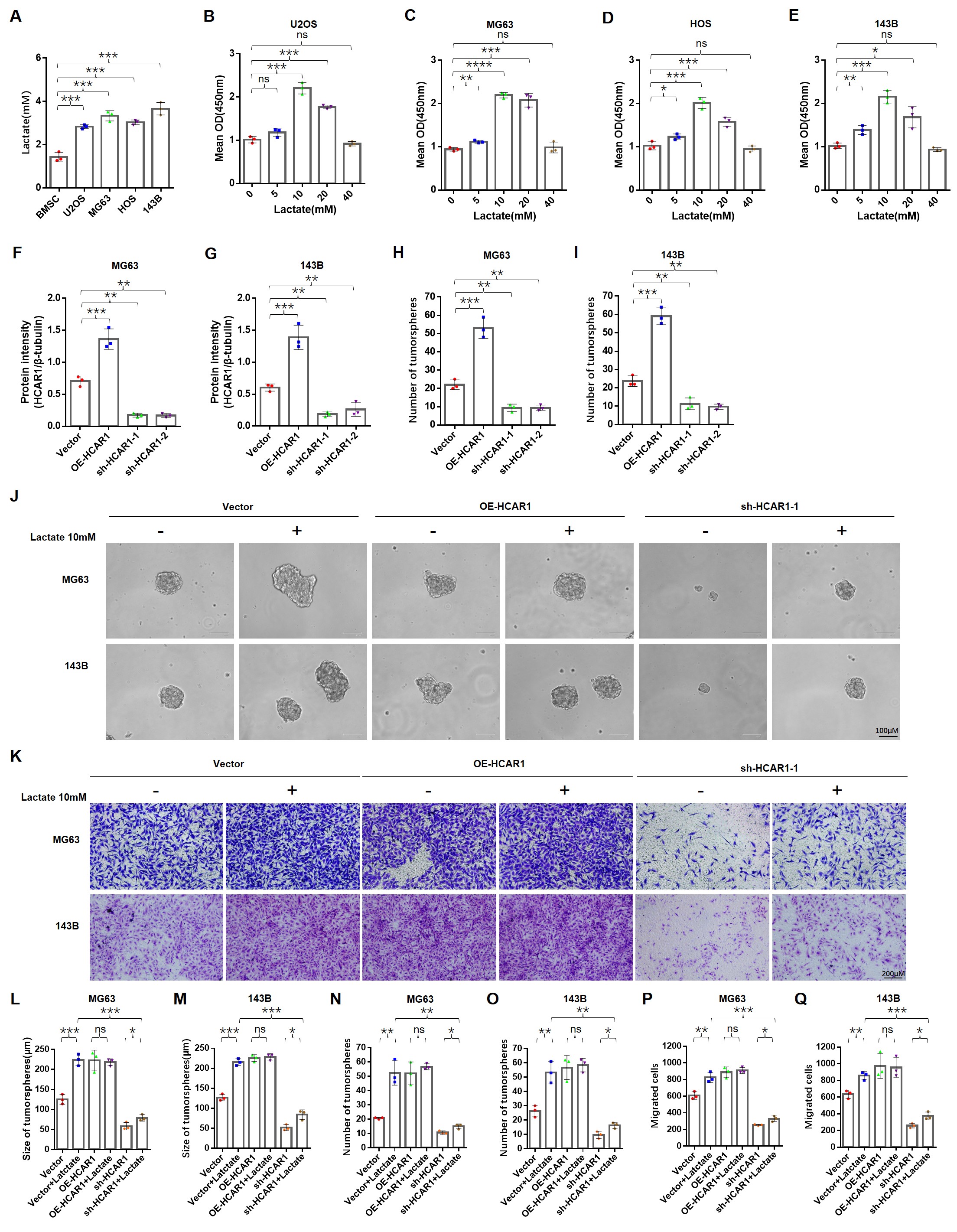


**S Figure. 3** Lactate was upregulated in OS and promoted cell proliferation and migration. **A** The level of extracellular lactate in bone marrow mesenchymal stem cells (BMSCs) and OS cells (including U2OS, MG63, HOS, 143B cell lines). **B-E** CCK8 assay performed to detect the effect of different concentrations of lactate on the proliferative activity of OS cells. **F, G** Validation of the protein expression of HCAR1 gain and loss in MG63 and 143B cells by western blot (also see Figure.2F, G). **H, I** The number of the tumor sphere-formation of the indicated OS cells featuring the over-expression or knockdown HCAR1 by soft agar clone formation assay (also see Figure.2J). **J, L-O** Soft agar clone formation assays performed as well as the size and number of the tumor sphere-formation of the effect of 10 mM lactate stimulation on the indicated cells. **K, P-Q** Transwell assays performed to evaluate the effects of 10 mM lactate stimulation on the migration and invasion of the indicated OS cells. Representative images and quantification of relative migrated cells are presented. Statistical analysis was performed using one-way ANOVA **(A-I)** and two-way ANOVA **(L-Q)**. Following by **post hoc testing** with **Tukey's Honestly Significant Difference (HSD) test (L-Q).** Error bars show means ± SD. *P < 0.05, **P < 0.01, ***P < 0.001, and ****P < 0.0001; NS, not significant. Scale bars, 100 μm or 200 μm.


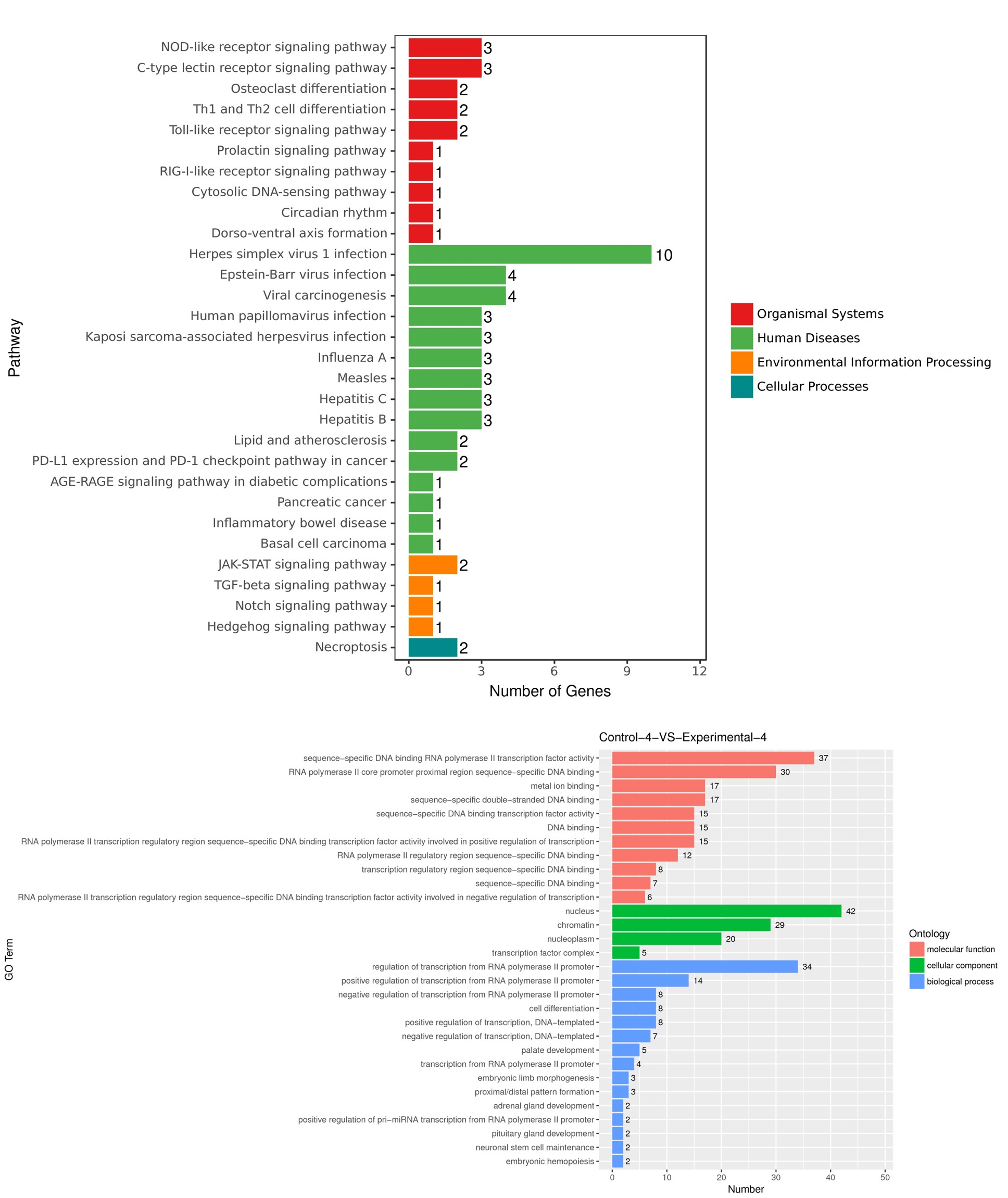


**S Figure. 4** KEGG and GO enrichment performed on the differential genes obtained by transcriptome sequencing.


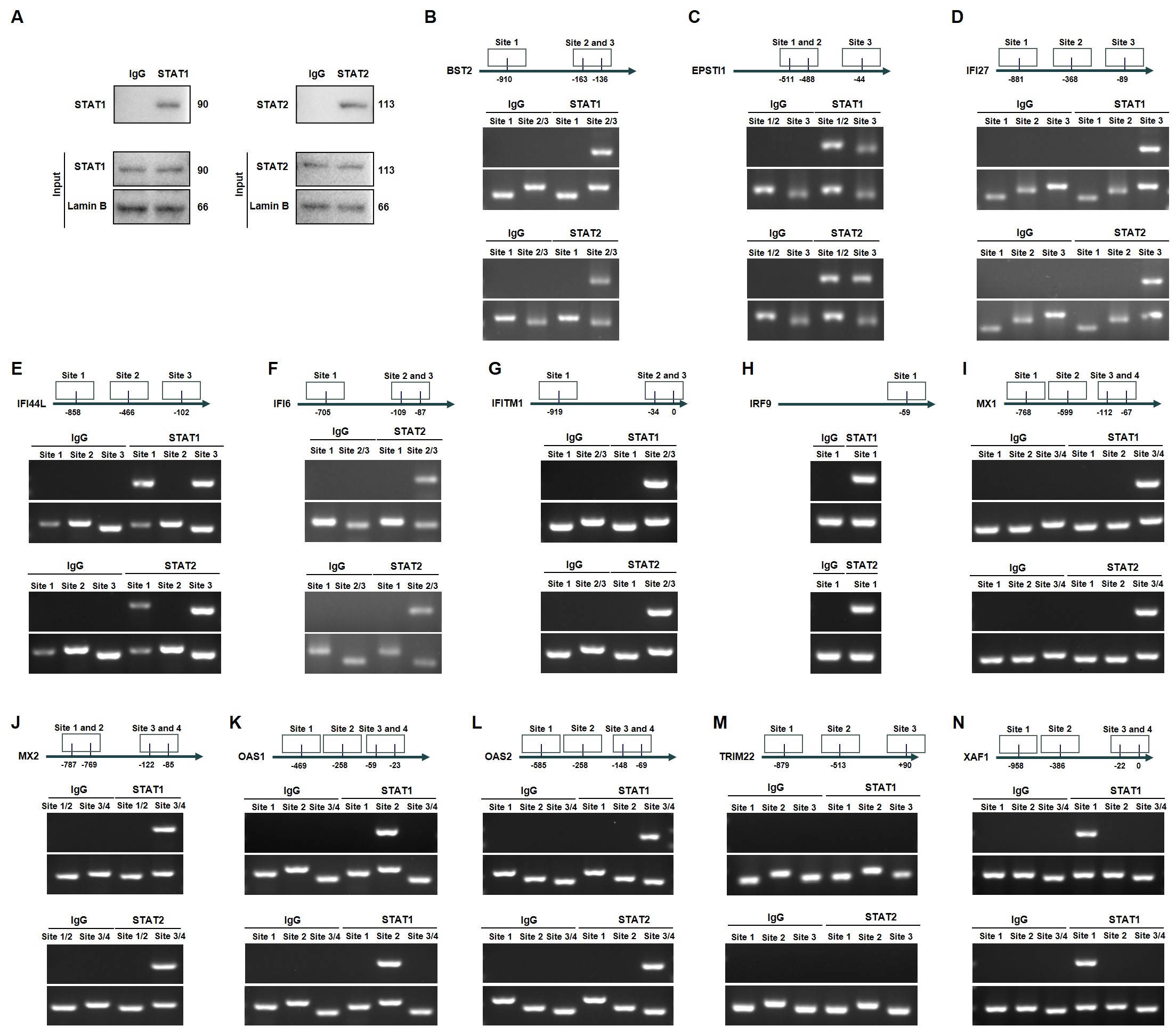


**S Figure. 5 Binding of STAT1/STAT2 to IFITM1, EPSTI1, TNFSF10, TRIM22, IFI27, BST2, IFI6, IFI44L, XAF1, OAS1, OAS2, IRF9, MX2, and MX1 was detected by ChIP-PCR. A STAT1/STAT2 in the precipitates was detected by Western blot. B-N The promoters of the aforementioned genes were analyzed by PCR.**


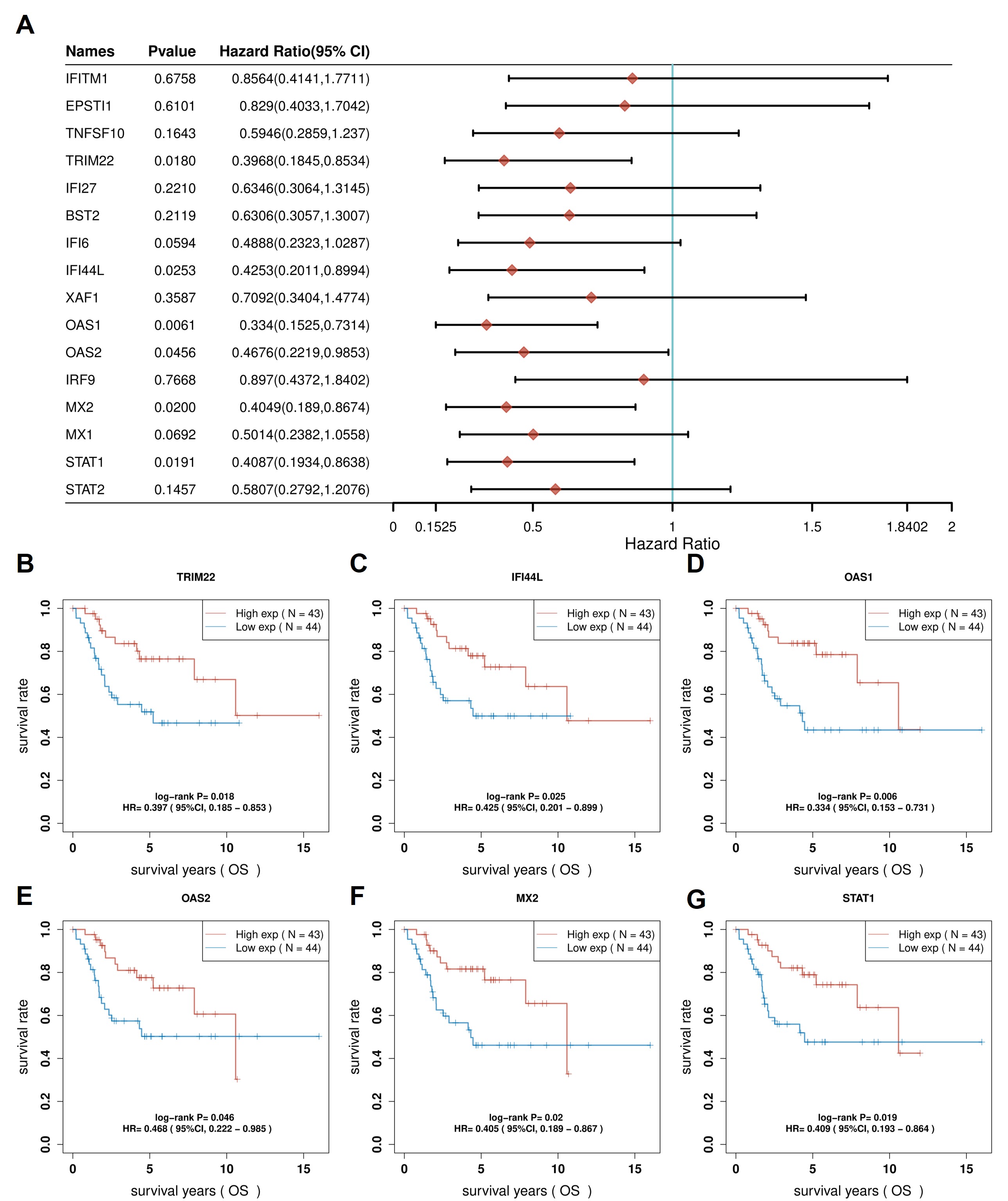


**S Figure. 6** The correlation between 14 differential genes and OS survival. **A The forest plot presents the univariate COX analysis of STAT1/2 and 14 differential genes in relation to osteosarcoma prognosis, along with their p-values, hazard ratios (HR), and confidence intervals. B-G** Kaplan-Meier curve of OS tissues, showing that among the 14 differential genes transcriptionally regulated by STAT1/2, 5 genes were positively associated with survival prognosis in OS based on TARGET database.


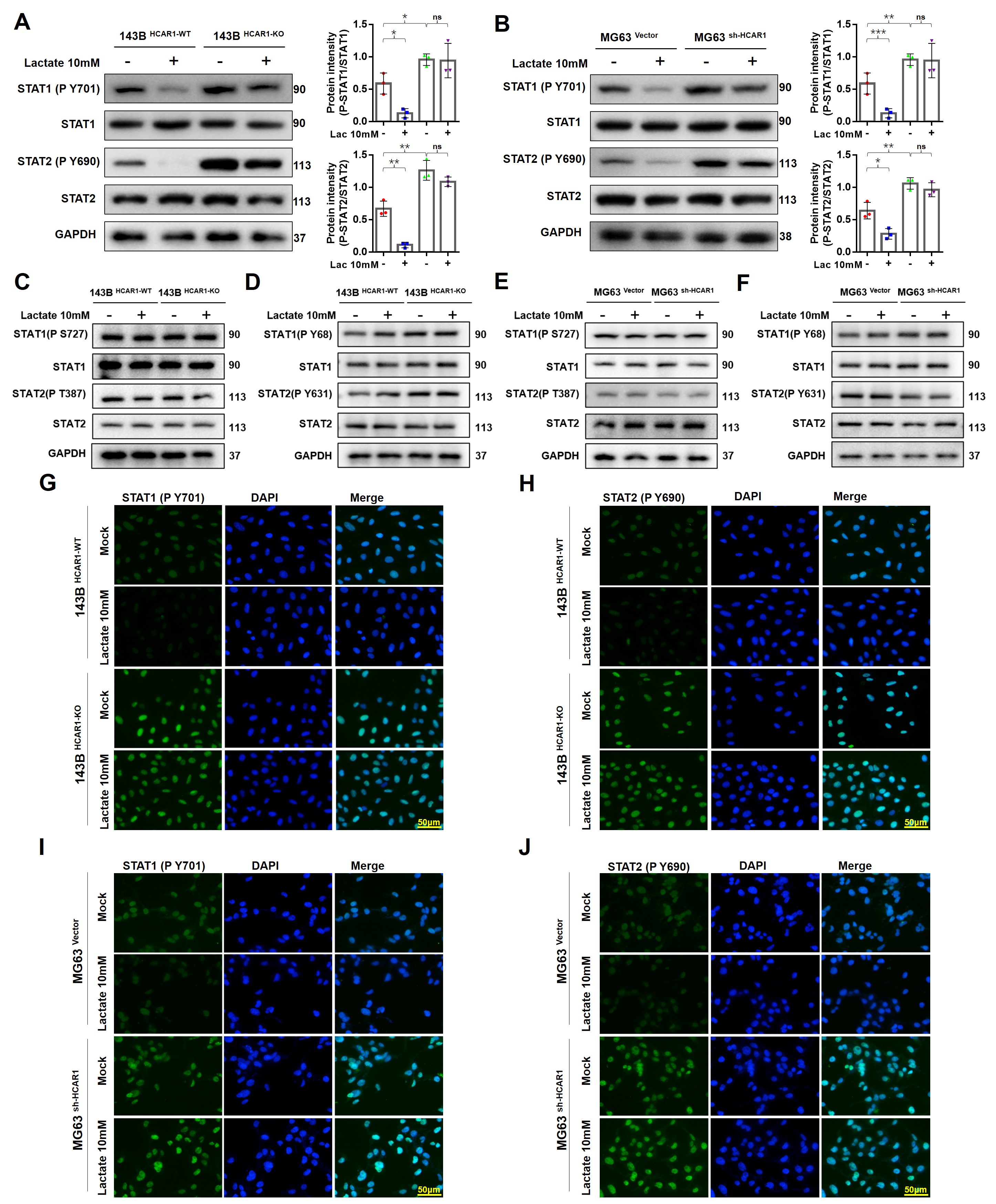


**S Figure. 7** Lactate/HCAR1 inhibits the phosphorylation of STAT1 Y701 and STAT2 Y690. **A-F** Western blots performed to detect the phosphorylation levels and overall levels of STAT1/2 expression in 143B and MG63 cells and evaluate the effect of lactate/HCAR1 on the indicated cells. **G-J** Immunofluorescence performed to detect the in-situ phosphorylation of STAT1/2 in OS cells, and assess the effects of lactate/ HCAR1 on the indicated cells. Statistical analysis was performed using two-way ANOVA, followed by **post hoc testing** with **Tukey's Honestly Significant Difference (HSD) test (A,B)**. Error bars show means ± SD. *P < 0.05, **P < 0.01, and ***P < 0.001; NS, not significant. Scale bars, 50 μm.


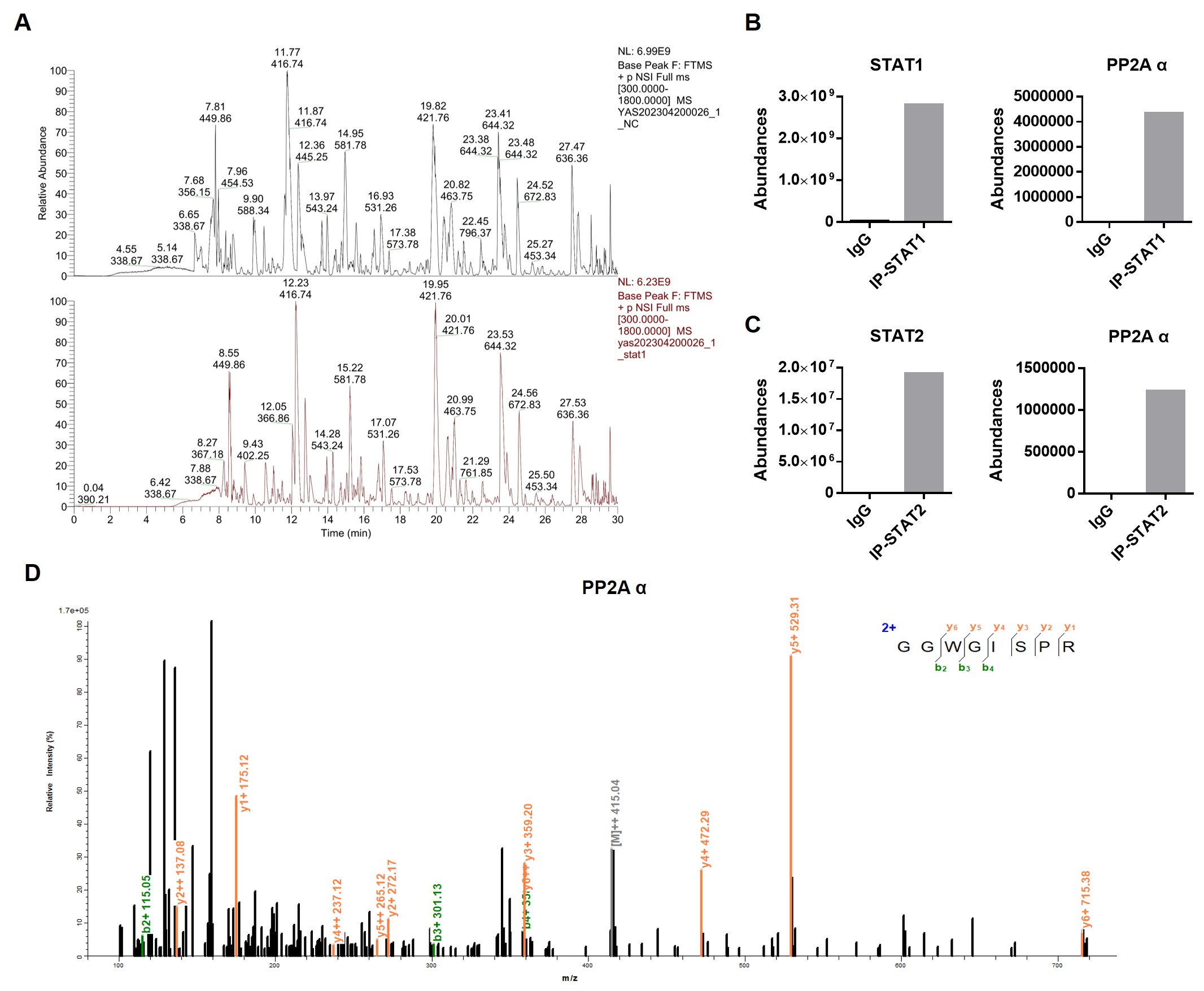


**S Figure. 8** LC-MS/MS assay of STAT1/2. **A-C** LC-MS/MS assay was performed to identify the potential proteins binding to STAT1/2, **with PPP1CA exhibiting high abundance among all specifically bound proteins. D** Specific peptide segments of PPP2CA using mass spectrometry.


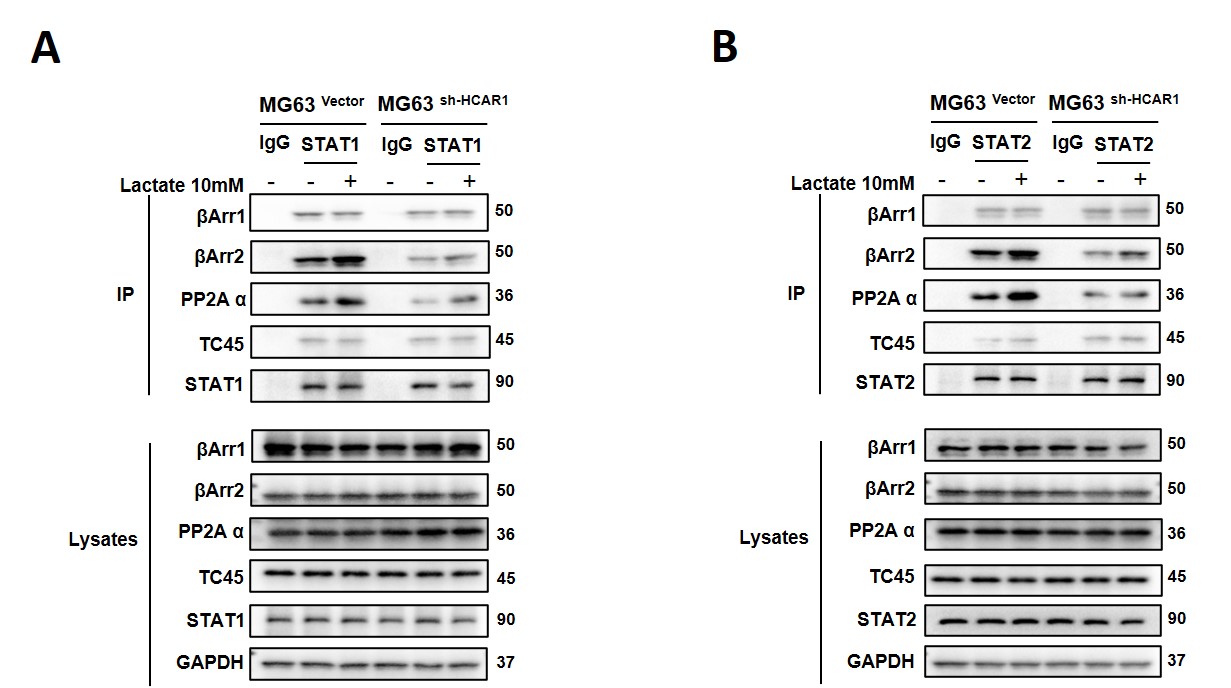


**S Figure. 9** Lactate activating HCAR1 enhances PP2Aα binding to STAT1/2 via β-Arrestin2 in MG63. **A** and **B**, Immunoprecipitation experiments performed to assess the effect of lactate/ HCAR1 on the endogenous interaction of STAT1/2 and PP2Aα, β-Arrestin1, β-Arrestin2, PP2Aα and TC45 of the indicated OS cells. MG63 cell lysates were subjected to immunoprecipitation with anti-STAT1/2, anti-β-Arrestin1, anti-β-Arrestin2, anti-PP2Aα or anti-TC45 antibody. The immunoprecipitates were then detected using the indicated antibodies.


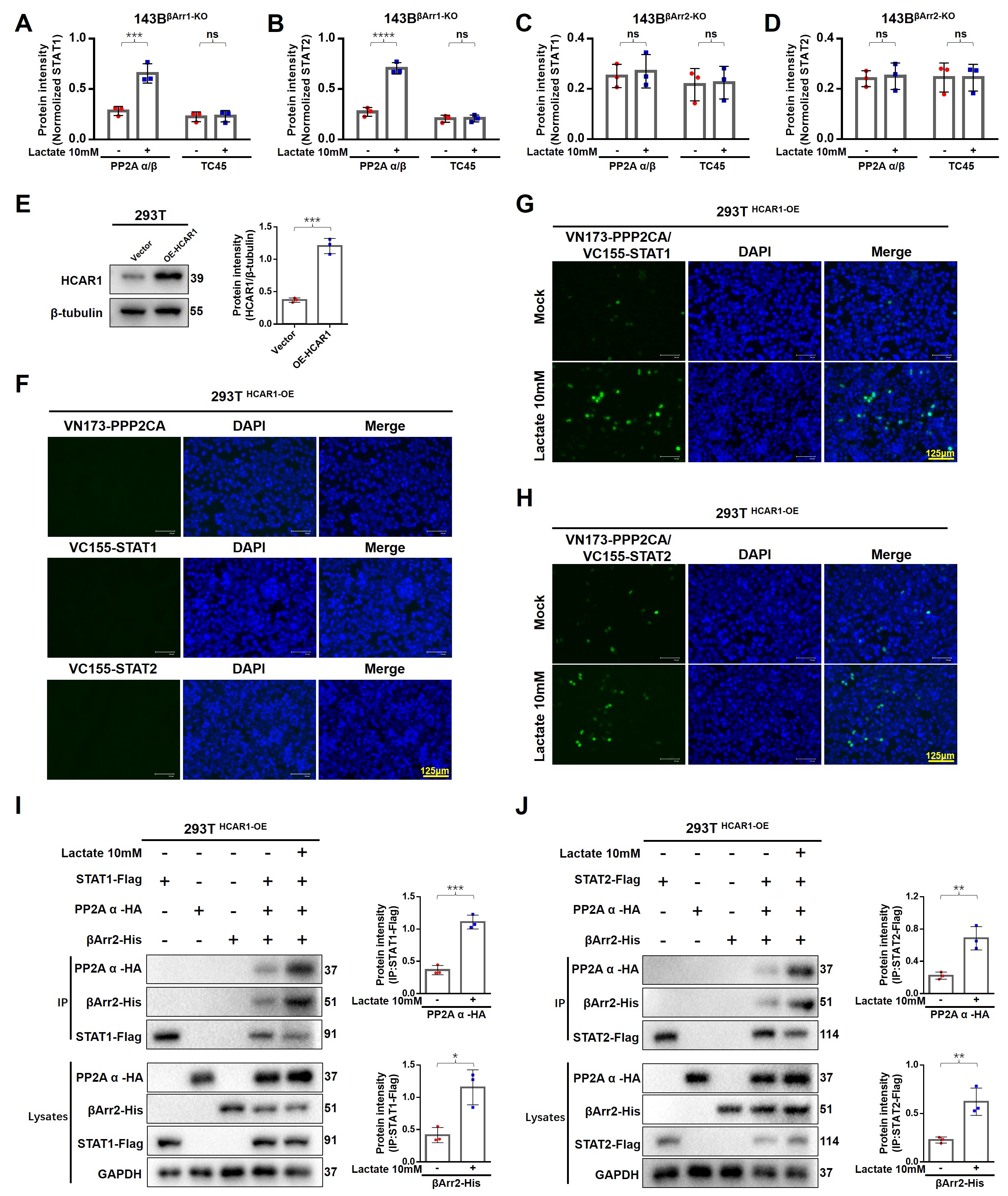


**S Figure. 10** 293T cells experiments were performed to detected the interaction of exogenous PP2A and STAT1/2. **A-D** Immunoprecipitation experiments performed to assess the effect of β-Arrestin1 and β-Arrestin2 on the endogenous interaction of STAT1/2 and PP2A α/β, TC45 of the indicated OS cells (also see Figure.5C-F). **E** Lentiviral infection performed to stably overexpress HCAR1 in 293T cells, and Western blot performed to verify the transfection efficiency. **F-H** Fluorescent bimolecular complementary plasmids, VN173-PP2Aα and VC155-STAT1/2 , co-transfected into 293T cells overexpressing HCAR1. **I, J** STAT1/2-Flag, PP2Aα-HA, and β-Arrestin2-His co-transfected into 293T cells overexpressing HCAR1. Immunoprecipitation experiments were performed to assess the effect of lactate/ HCAR1 on the exogenous interaction of STAT1/2 and PP2Aα, β-Arrestin2 in 293T cells. Statistical analysis was performed using unpaired two-way ANOVA, followed by **post hoc testing** with **Tukey's Honestly Significant Difference (HSD) test (A-D)**, and one-way ANOVA **(E, I, J)**. Error bars show means ± SD. *P < 0.05, **P < 0.01, ***P < 0.001, and ****P < 0.0001; NS, not significant. Scale bars, 125 μm.

**
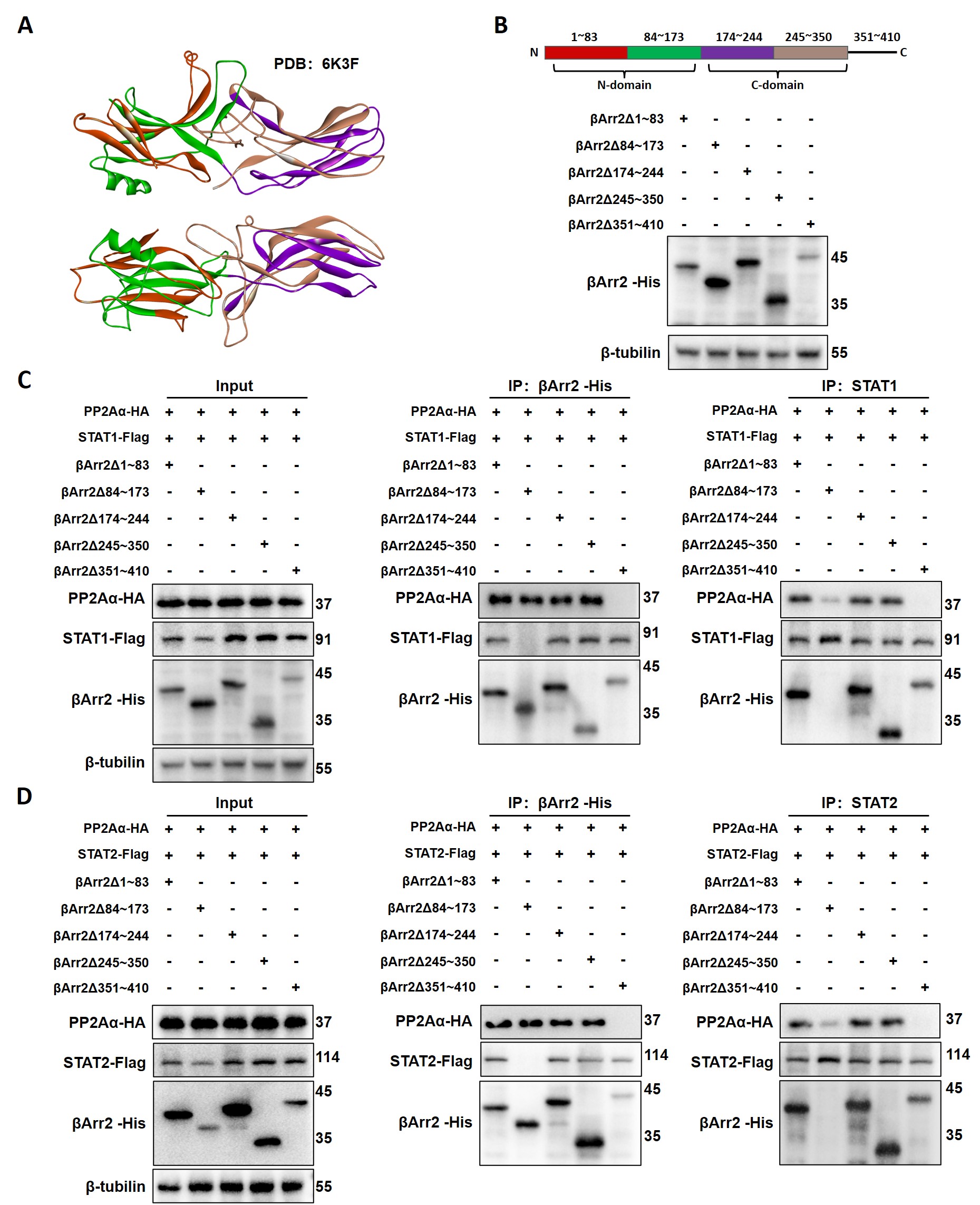
**

**S Figure. 11** STAT1/2 binds to the α-helical region within the N-domain of β-Arrestin2, while PP2Aα interacts with its C-terminus. **A** **Schematic Structure of β-Arrestin2 (**PDB6K3F). **B** **Schematic diagrams of β-Arrestin2 truncated forms and western blot detection of β-Arrestin2-His expression**. **C-D** STAT1/2-Flag, PP2Aα-HA, and β-Arrestin2-His with t**runcated mutantation were** co-transfected into 293T cells overexpressing HCAR1. **Binding sites between STAT1/2, PP2Aα, and β-Arrestin2 were assessed by Co-IP assays.**

**
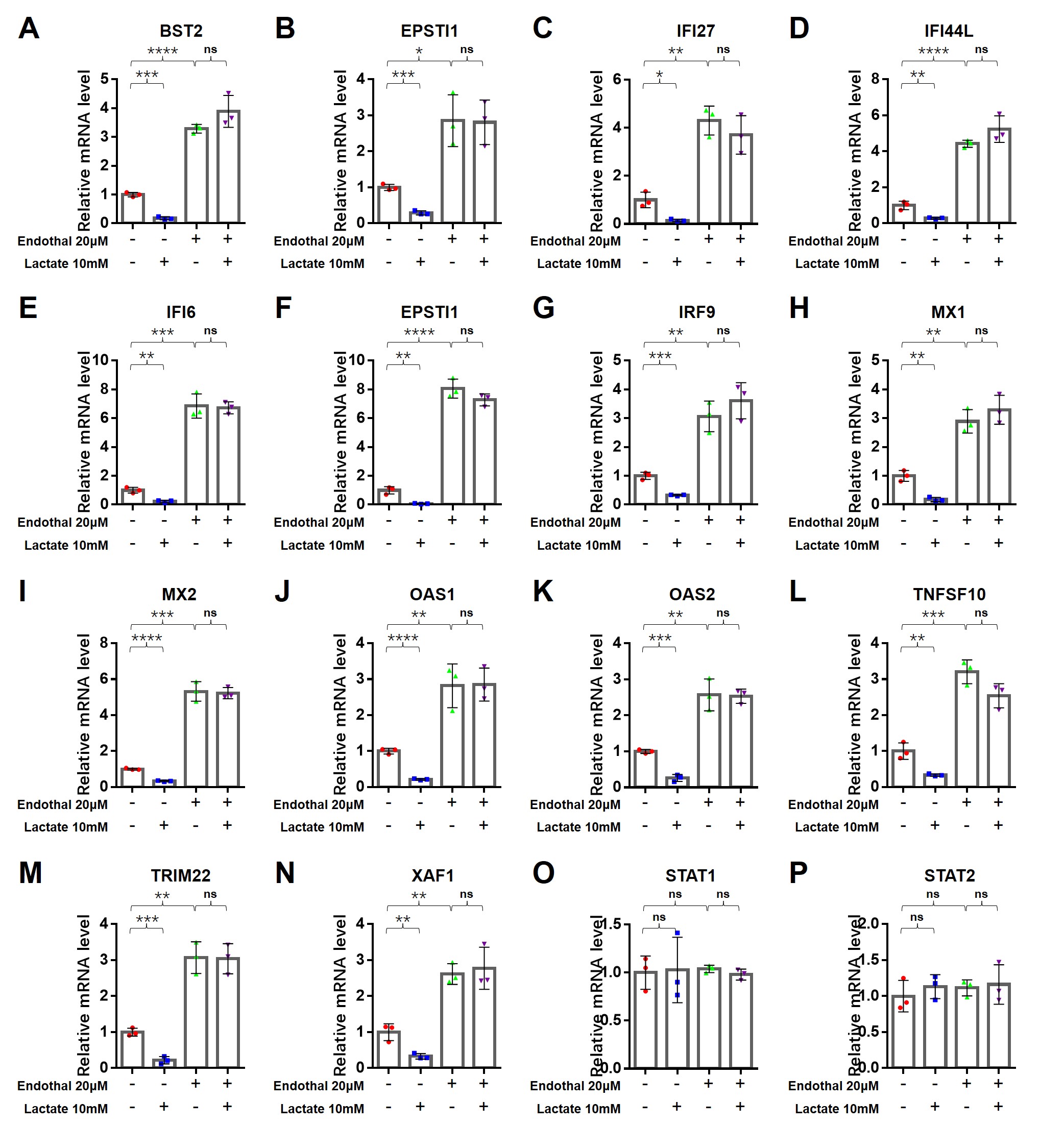
**

**S Figure. 12** Validation of the effect of Endothall treatment on the mRNA levels of STAT1/2 and its target genes by qRT-PCR. Statistical analysis was performed using two-way ANOVA, followed by **post hoc testing** with **Tukey's Honestly Significant Difference (HSD) test**. Error bars show means ± SD . *P < 0.05, **P < 0.01, ***P < 0.001, and ****P < 0.0001; NS, not significant.


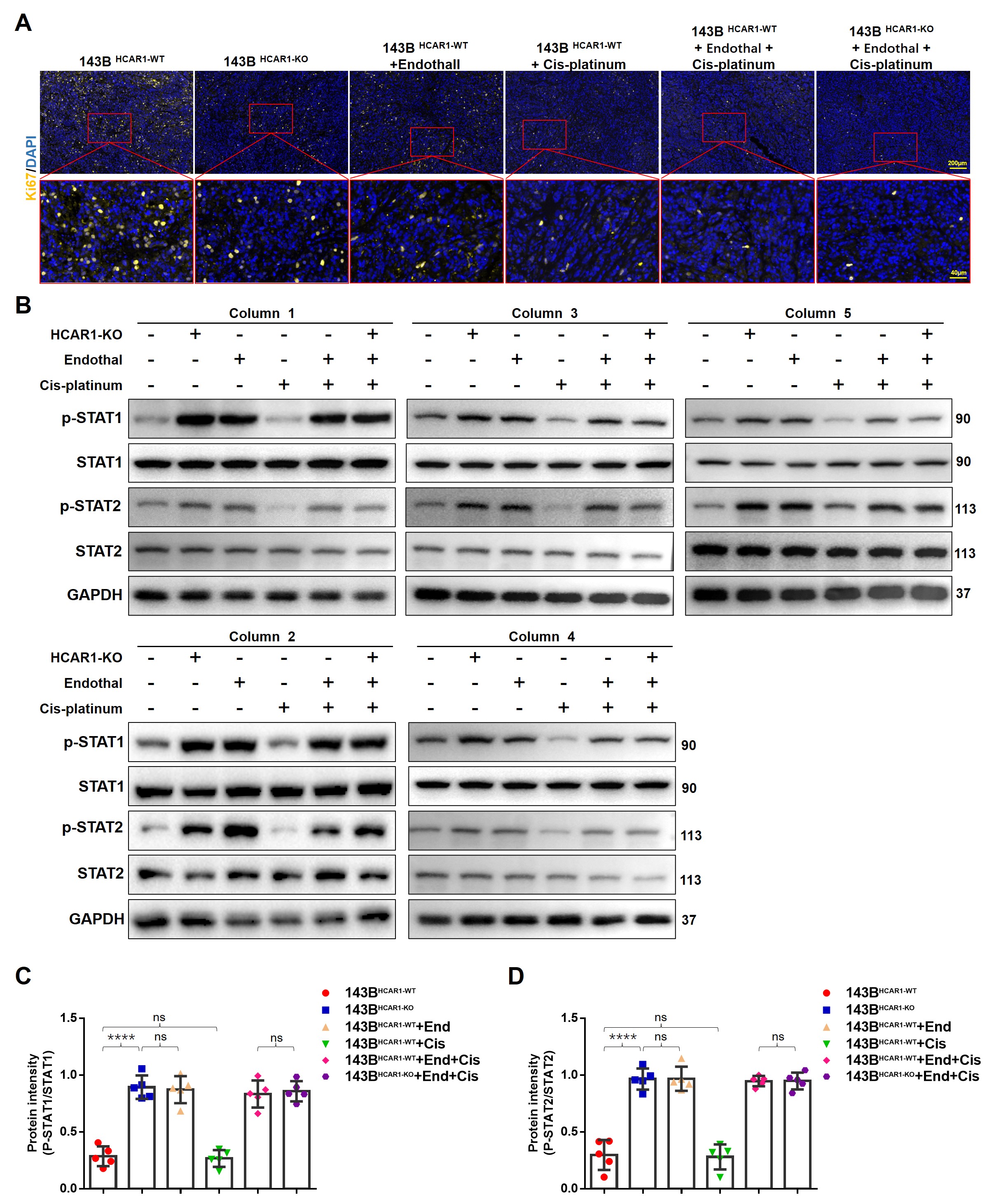


**S Figure. 13** In vivo experiment, HCAR1 knockout or Endothall treatment inhibited OS proliferation and STAT1/2 dephosphorylation. **A** The expression of Ki67 in each group tumors detected by immunohistochemistry. **B** The phosphorylation levels and overall levels of STAT1/2 in each group tumors detected by Western Blot. **C-D** Protein content of the phosphorylated STAT1/2 in the tumors calculated for each group and subjected to statistical analysis. Statistical analysis was performed using multifactor ANOVA, followed by **post hoc testing** with **Tukey's Honestly Significant Difference (HSD) test (C, D)**. Error bars show means ± SD. ****P < 0.0001; NS, not significant. Scale bars, 40 μm or 200 μm.


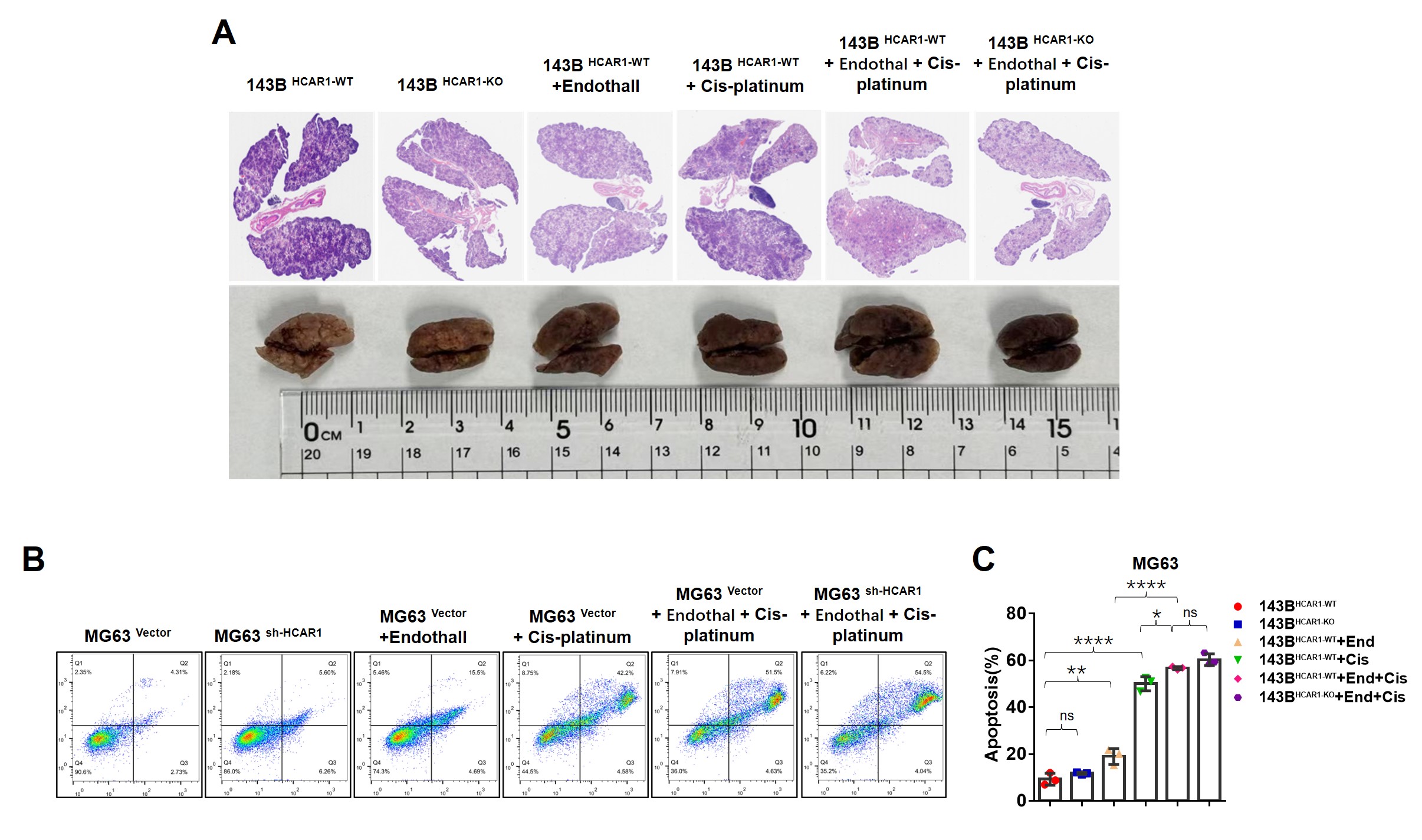


**S Figure. 14** **H&E-stained pathological sections of murine lung tissues and flow cytometric apoptosis analysis of MG63 cells.** **A** **Pathology and H&E-stained histological sections of lung tissues from each experimental group**. **B-C** Using cisplatin as a reference (positive drug), the killing efficacy of HCAR1 knockout or Endothall on MG63 cells evaluated by flow cytometry. Statistical analysis was performed using multifactor ANOVA, followed by **post hoc testing** with **Tukey's Honestly Significant Difference (HSD) test** (**C**). Error bars show means ± SD. *P < 0.05, **P < 0.01****P < 0.0001; NS, not significant.
