# Supplementary material for: Lactate Activates the HCAR1/β‐Arrestin2/PP2A Signaling Axis to Mediate STAT1/2 Dephosphorylation and Drive Osteosarcoma Progression
Source: Adv Sci (Weinh). 2025 Sep 16;12(45):e06214. doi: 10.1002/advs.202506214 (PMC12677626; doi:10.1002/advs.202506214)

**Supplementary Information**

**
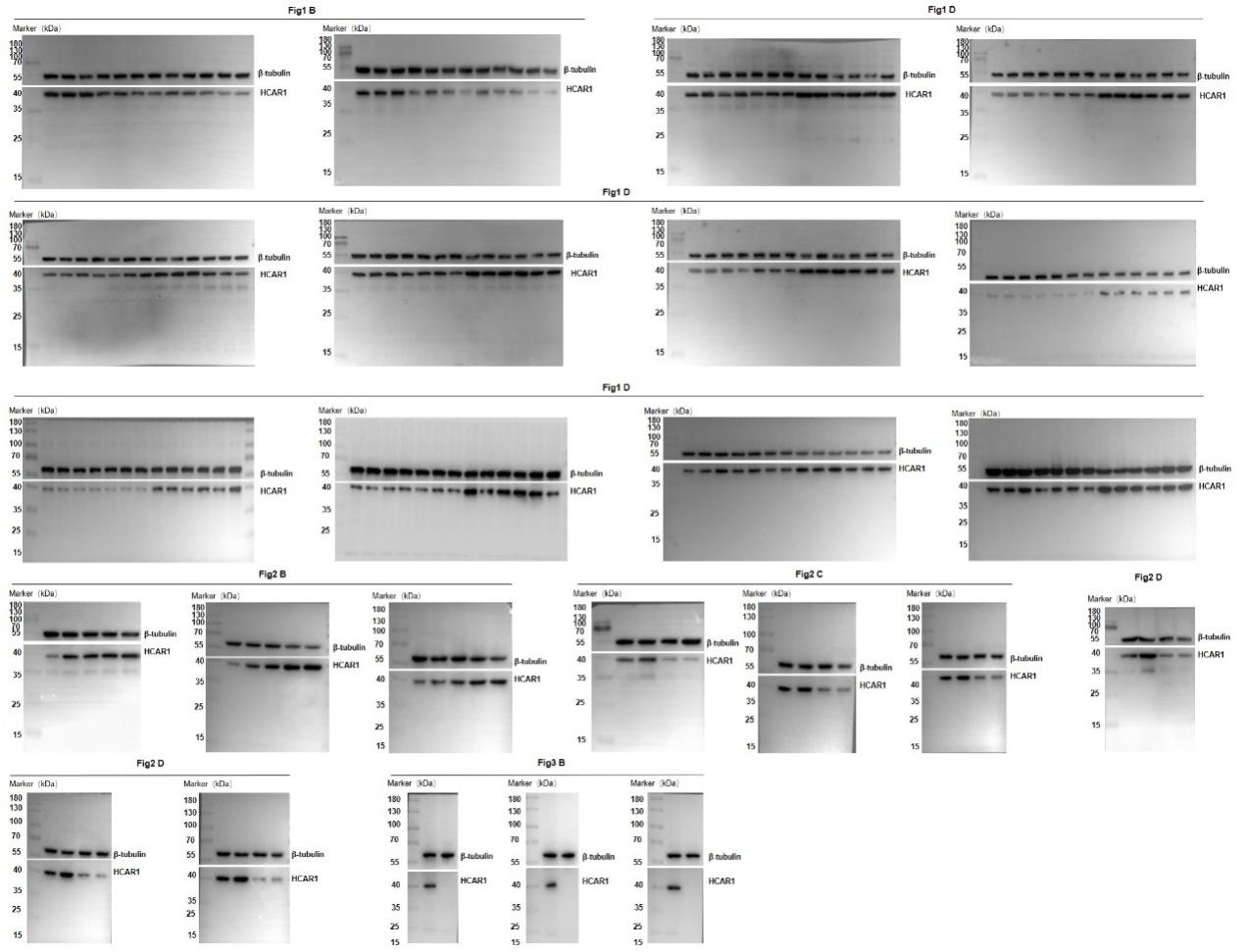
**

1. Original, uncropped images of blot results (Fig. 1-3).
2.
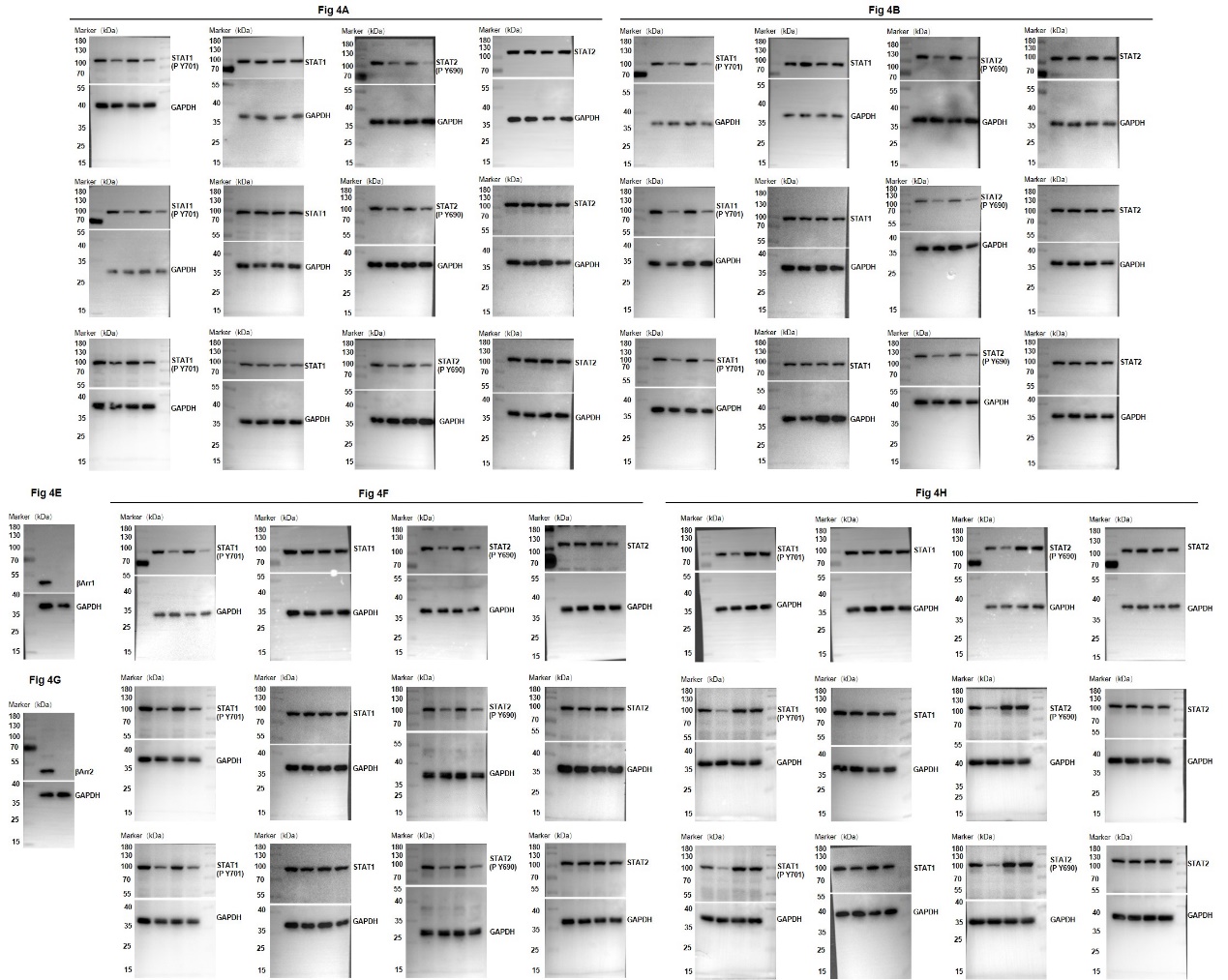
Original, uncropped images of blot results (Fig. 4).
3.
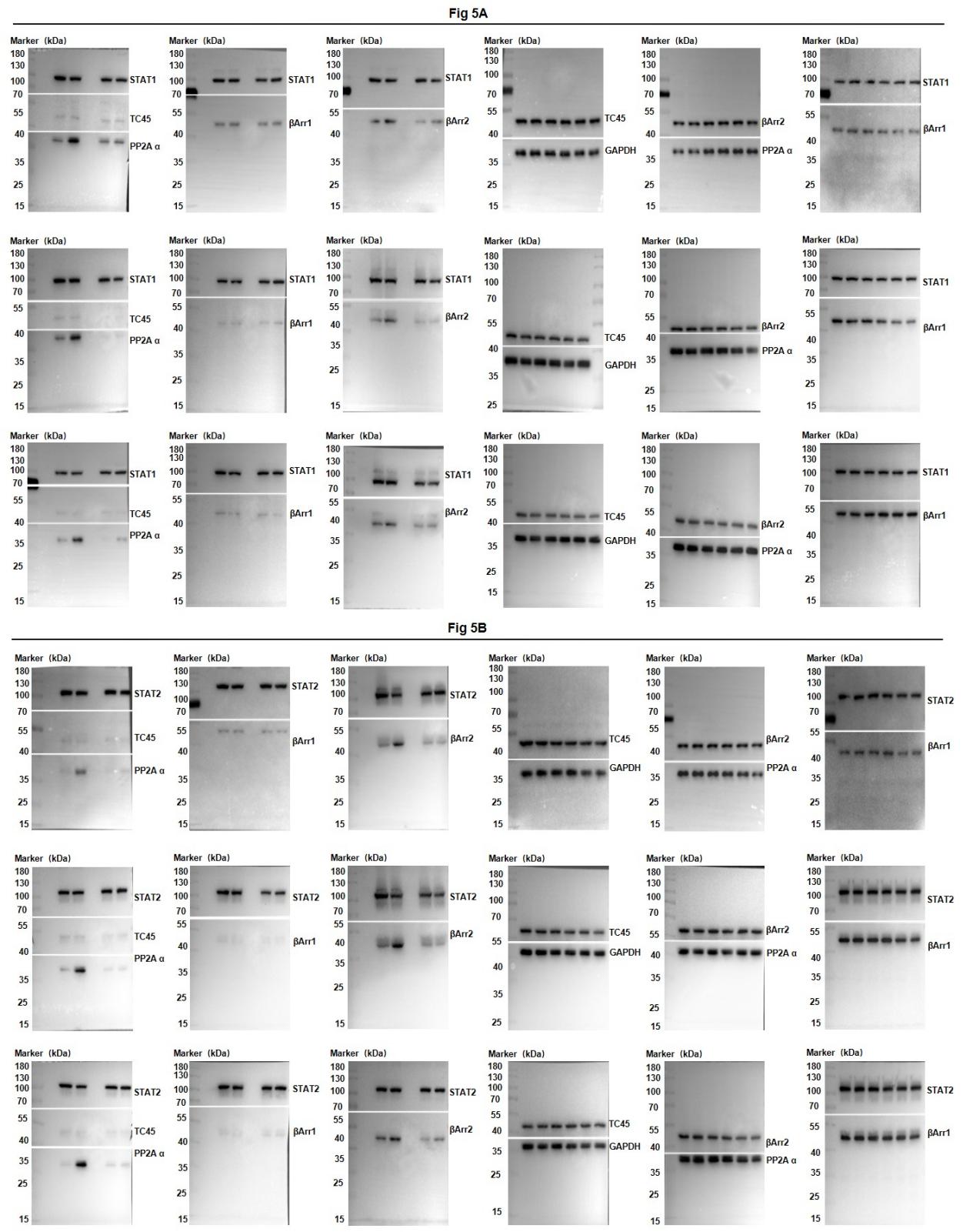
Original, uncropped images of blot results (Fig. 5A-B).
4.
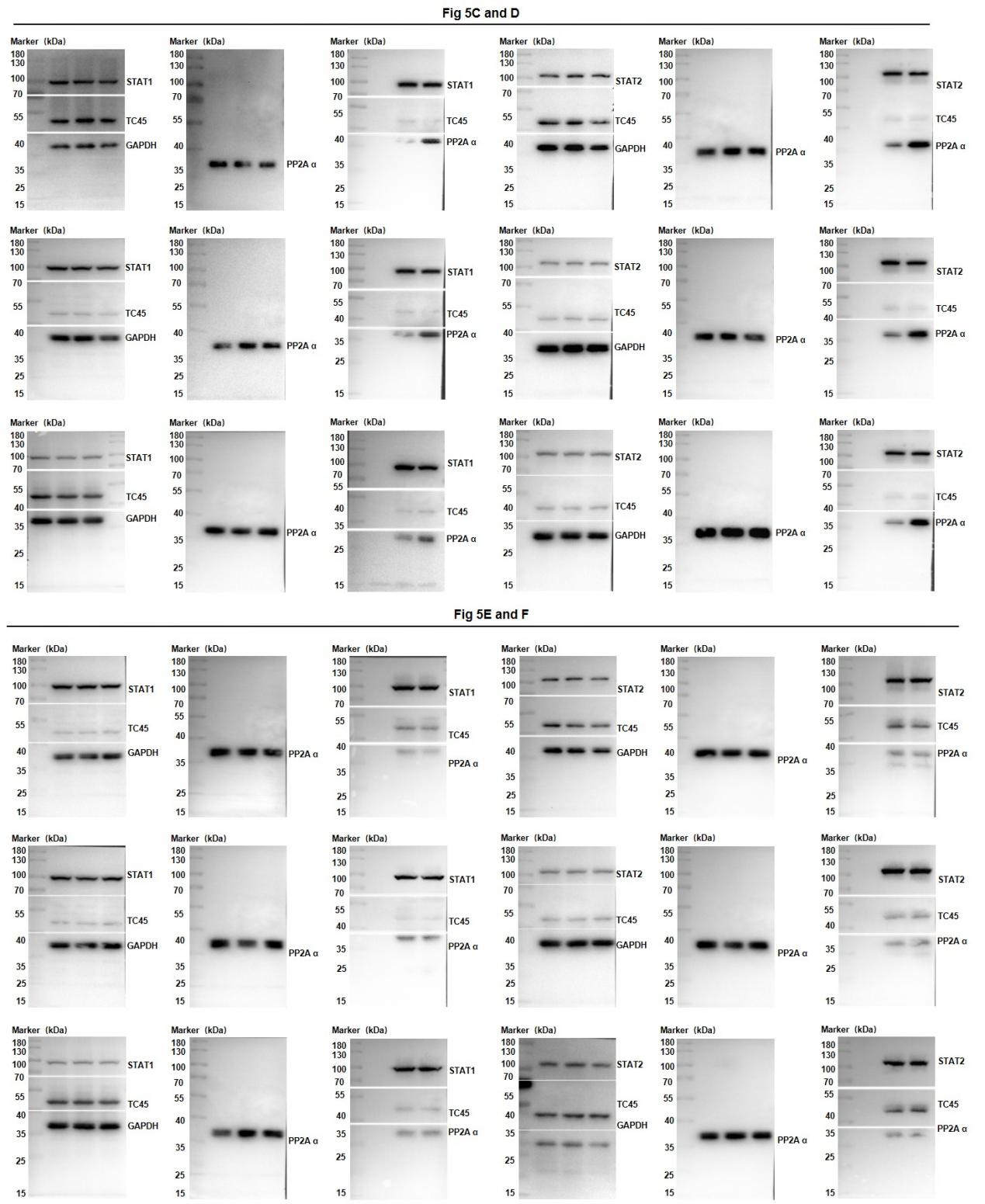
Original, uncropped images of blot results (Fig. 5C-F).
5.
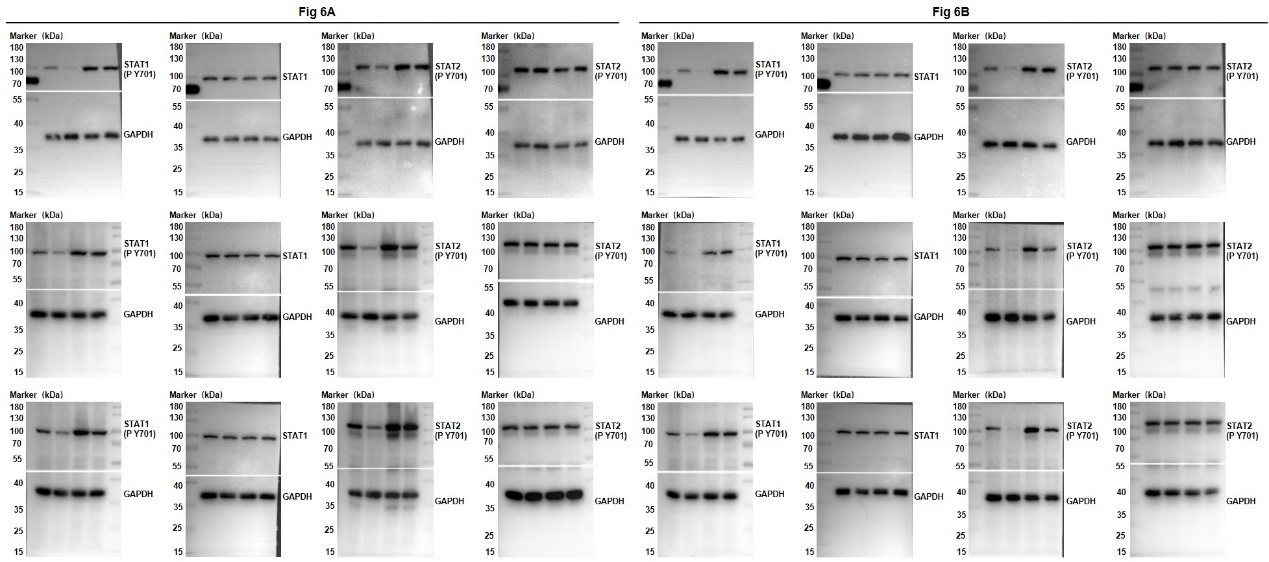
Original, uncropped images of blot results (Fig 6).


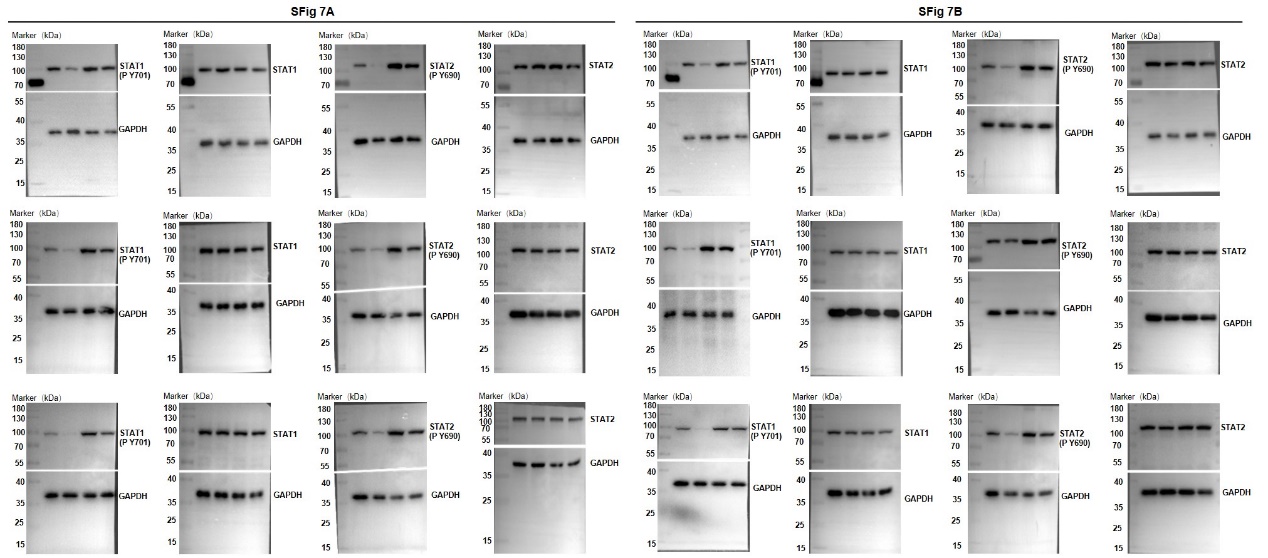


1. Original, uncropped images of blot results (SFig 7).
2.
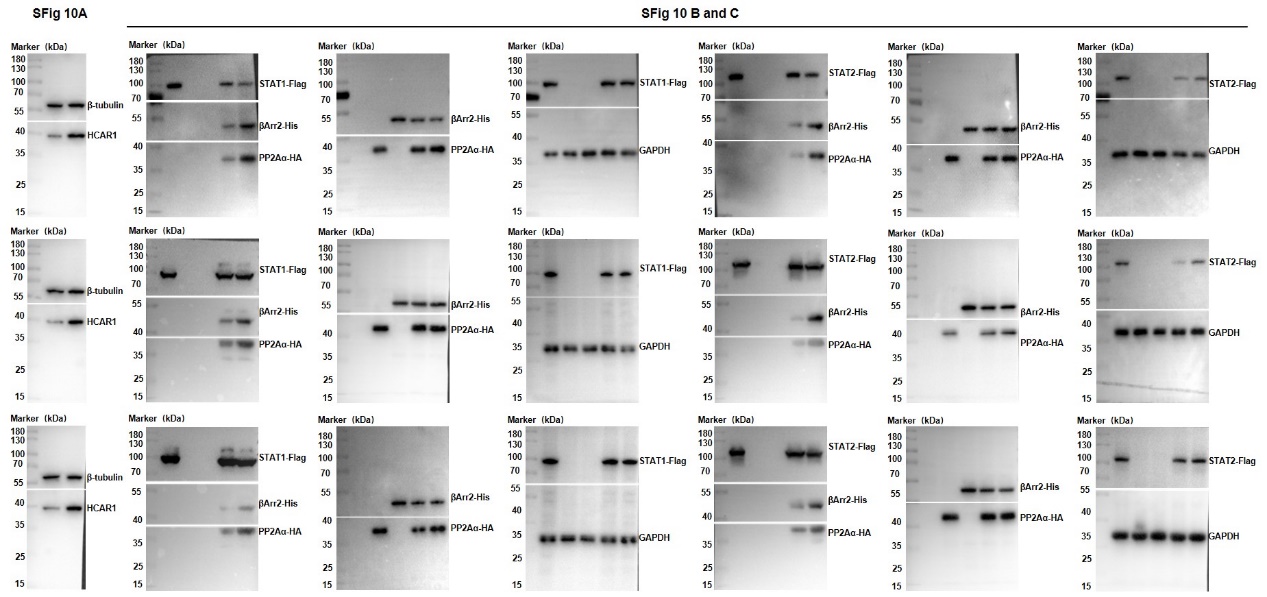
Original, uncropped images of blot results (SFig 10).
3.
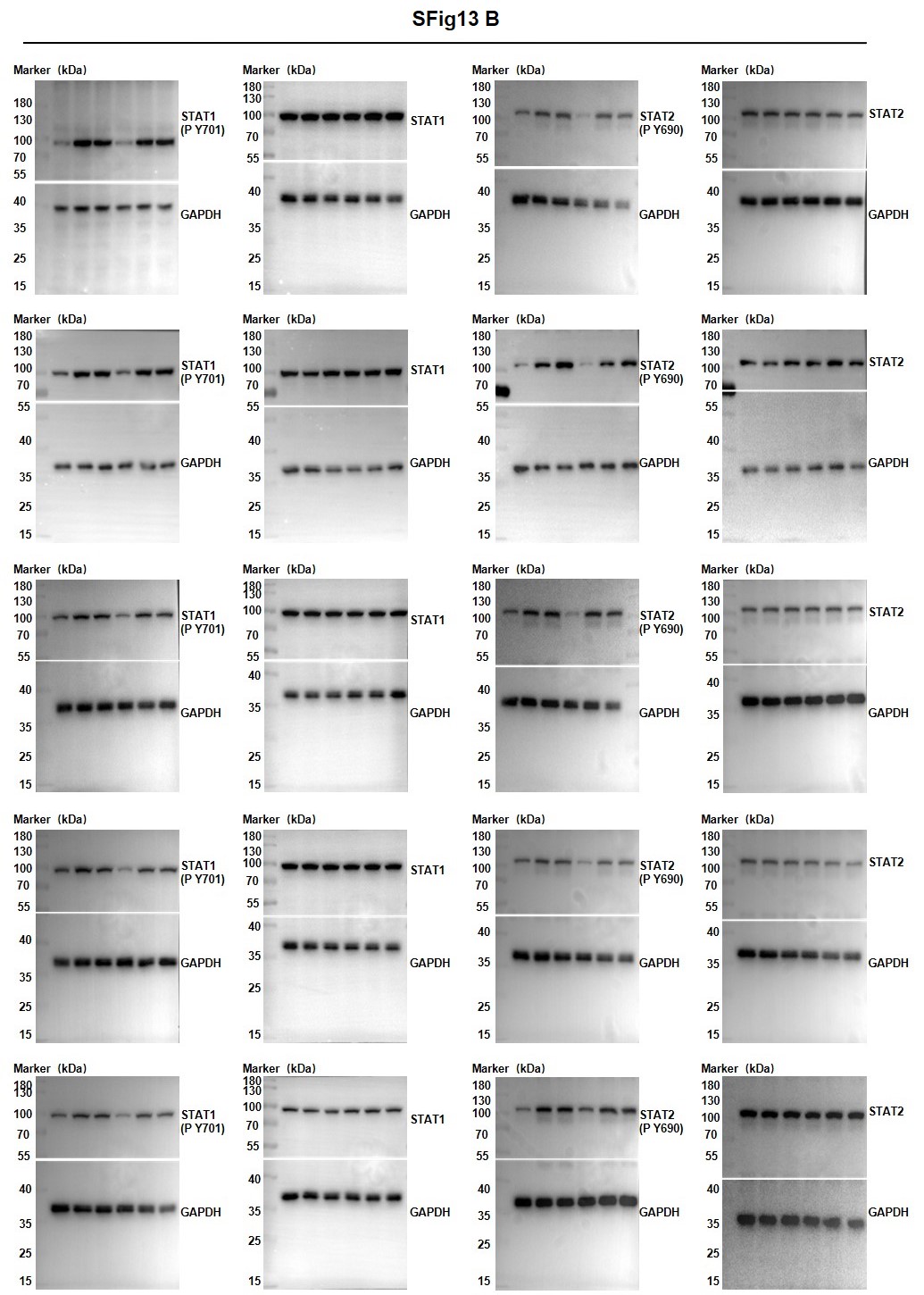
Original, uncropped images of blot results(SFig 13).
4.
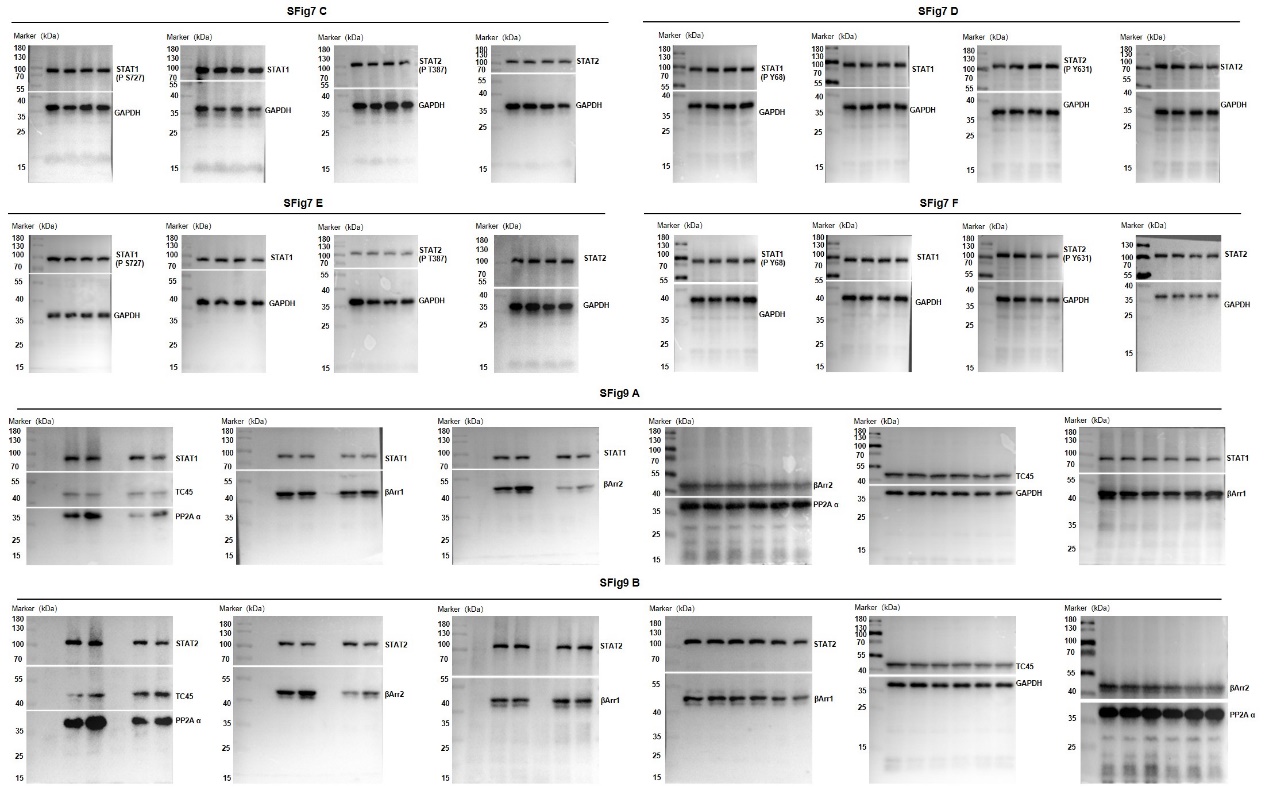
Original, uncropped images of blot results (SFig 7C-F and SFig 9A-B).
5. Original, uncropped images of blot results (SFig 7C-F and SFig 9A-B).
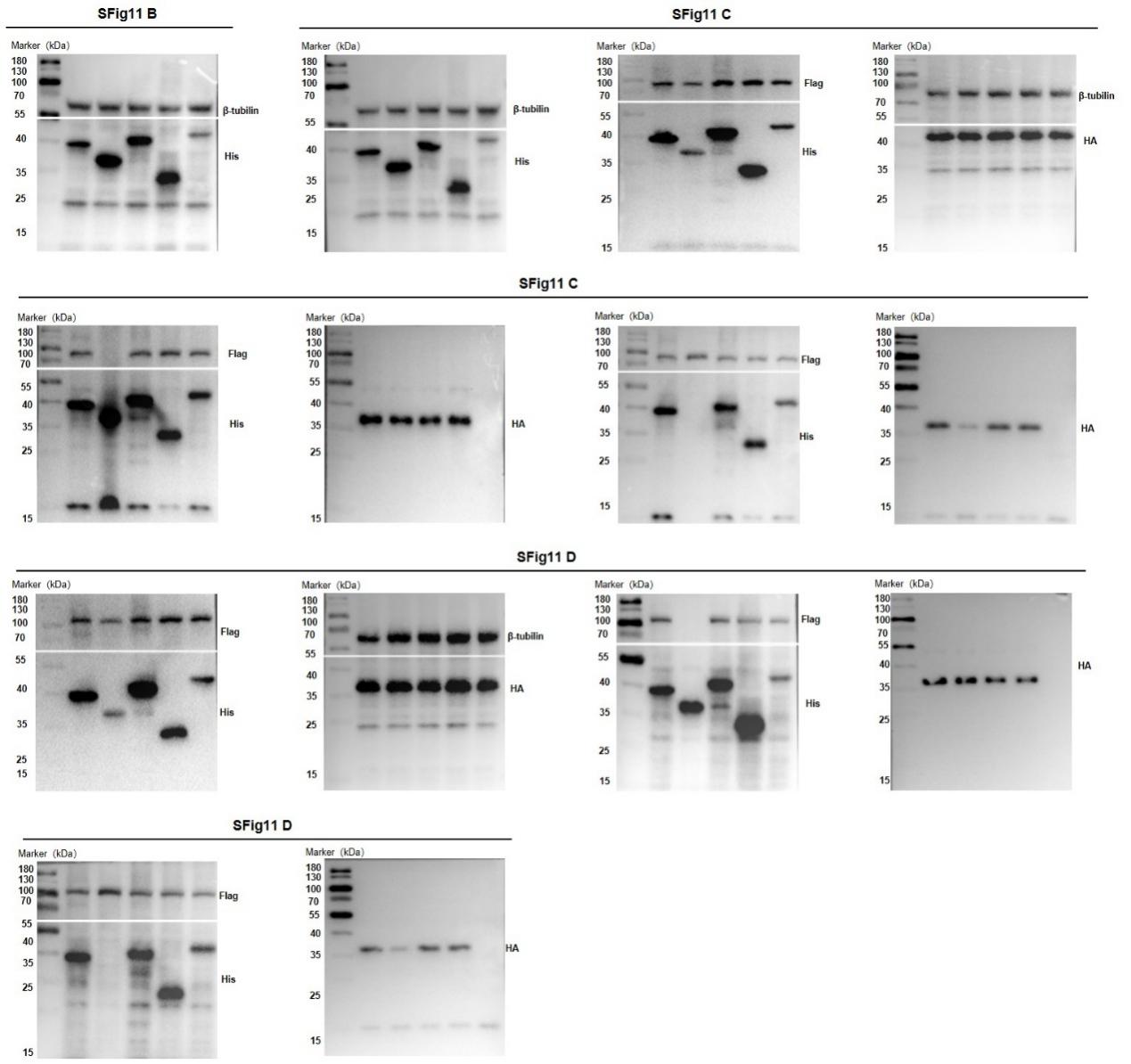

Supplement: Supplementary file 3 — Supporting Information [file ADVS-12-e06214-s006.zip › Additional file 8.docx]
